# Lohnt sich AIT? Wirtschaftlichkeitsbetrachtung der allergenspezifischen Immuntherapie (AIT) für den niedergelassenen Hals-Nasen-Ohren(HNO)-Arzt

**DOI:** 10.1007/s00106-024-01542-8

**Published:** 2025-02-03

**Authors:** U. Reineke, C. Cappello

**Affiliations:** 1 HNO- und Allergiezentrum Herford, Berliner Straße 6–8, 32052 Herford, Deutschland; 2 Bergstr. 17, 83059 Kolbermoor, Deutschland

**Keywords:** Ärztliches Honorar, Berechnungsmodell, Stundenhonorar, SCIT, SLIT, Physician’s fee, Calculation model, Hourly rate, Subcutaneous immunotherapy, Sublingual immunotherapy

## Abstract

Allergien haben für Patienten, aber auch für die Kostenträger und die Volkswirtschaft eine enorme Bedeutung. Die Wirksamkeit und die Kosteneffektivität der allergenspezifischen Immuntherapie (AIT) gegenüber symptomatischen Behandlungen wurden bereits in verschiedenen Studien belegt. In Deutschland sind Allergiepatienten jedoch unterversorgt. Ziel dieser Arbeit ist die Untersuchung der Honorierung der allergologischen Diagnostik und AIT für niedergelassene Hals-Nasen-Ohren(HNO)-Ärzte. Eine Modellberechnung wurde für die Vergütung allergologischer Leistungen für zwölf Kassenärztliche Vereinigungen (KV) auf Basis des einheitlichen Bewertungsmaßstabes (EBM) und der KV-spezifischen Regelungen für das Jahr 2022 durchgeführt [20, 22]. Die Honorierung wurde sowohl gemäß der Werte im EBM als auch mit einem abgesenkten Punktwert kalkuliert. Die Vergütung wurde quartalsweise, für eine dreijährige Therapie und pro Stunde für subkutane Immuntherapien (SCIT) und sublinguale Immuntherapien (SLIT) analysiert und in Relation zum jeweiligen durchschnittlichen Honorar pro Behandlungsfall (DHPB) gesetzt. Bei Einhaltung des Budgets liegt die Honorierung eines HNO-Arztes in jeder KV bei einer SCIT über dem DHPB. Dies ist in den meisten KV auch dann der Fall, wenn der Überschuss zum Fallwert nur mit 15 % honoriert wird. Das Honorar bei einer dreijährigen SLIT ist in allen KV geringer als das DHPB. Die allergologische Diagnostik und die AIT rechnen sich für HNO-Praxen. Die Fortsetzungsbehandlung zeigt ein positives Aufwand-Ertrag-Verhältnis und unterstützt somit die Forderung nach einer dreijährigen Therapietreue.

**Zusatzmaterial online:**

Die Online-Version dieses Beitrags (10.1007/s00106-024-01542-8) enthält zusätzliche Informationen.

## Abstract (en)

The burden of allergic diseases is significant for patients, payers in the healthcare system, and the political economy. Numerous studies have demonstrated the efficacy and cost-effectiveness of allergen immunotherapy (AIT) compared to symptomatic treatments. However, allergic patients in Germany often do not receive adequate treatment. This study aims to investigate the reimbursement of allergy diagnostics and AIT for ear, nose, and throat (ENT) physicians in private practice. A model calculation was conducted to assess the reimbursement of allergology services across 12 statutory health insurance associations (*Kassenärztliche Vereinigungen*, KV) based on the Physician Fee Schedule (*Einheitlicher Bewertungsmaßstab*, EBM) and KV-specific regulations for the year 2022 [20, 22]. Reimbursement was calculated according to EBM values and with a reduced point value. Quarterly reimbursement for a 3-year therapy and hourly rates for subcutaneous immunotherapy (SCIT) and sublingual immunotherapy (SLIT) were analyzed and compared with the average reimbursement per case (*durchschnittlicher Honorarumsatz je Behandlungsfall*, DHPB). Adherence to the budget resulted in ENT physicians being reimbursed above the DHPB for SCIT in every KV. This remained true for most KVs, even when the excess to the case value was only rewarded at 15%. Reimbursement for a 3-year SLIT was lower than the DHPB across all KVs. Allergy diagnostics and AIT represent economically advantageous options for ENT practices. Maintenance therapy demonstrates a positive cost–benefit ratio, supporting the call for a consistent 3‑year treatment regimen.

Allergische Erkrankungen, wie z. B. die allergische Rhinitis (AR), haben für Patienten^1^, die Kostenträger und die Volkswirtschaft insgesamt eine große Bedeutung [24, 27]. Nicht nur die direkt durch die Erkrankung verursachten Kosten, sondern auch die indirekten sowie die intangiblen Kosten (Kosten für krankheitsbedingte Einschränkungen, wie z. B. Schmerz, oder die reduzierte Lebensqualität) belasten die Betroffenen und die Gesundheitssysteme [4–6, 23, 27]. Allergien gelten als Ursache für 1 % aller durch Krankheit und Tod verlorenen Lebensjahre in Deutschland und liegen damit auf gleichem Niveau wie Diabetes mellitus [23, 30]. Jede zehnte Krankschreibung in Deutschland wird einem allergischen Krankheitsbild zugeordnet [23].

Als einzige kausale Therapie steht die allergenspezifische Immuntherapie (AIT) zur Verfügung [27]. Ihre krankheitsmodifizierende Eigenschaft wurde sowohl für die subkutane (SCIT) als auch für die sublinguale allergenspezifische Immuntherapie (SLIT) in klinischen Studien, Metaanalysen und Langzeitbeobachtungen in der Praxis („real-world evidence“, RWE) nachgewiesen [25, 27, 32]. Um einen Therapieerfolg mit Langzeiteffekt zu erhalten, wird empfohlen, die Therapie mindestens drei Jahre durchzuführen [26, 27, 31]. Einige Studien zeigen auch einen präventiven Effekt der AIT und lassen auf die Verhinderung eines Etagenzuwachses zum allergischen Asthma (AA) im Sinne einer Krankheitsmodifikation schließen [27].

Die Kosteneffektivität der AIT gegenüber einer rein symptomatischen Behandlung wurde bereits in verschiedenen Studien belegt [2–4, 23, 29]. Brüggenjürgen et al. analysierten erstmalig 2021 die Kosten-Effektivität auf der Grundlage von RWE-Daten zur Therapieadhärenz für Deutschland und kamen zu dem Schluss, dass die AIT im Vergleich zur symptomatischen Behandlung eine kosteneffektive Behandlungsoption für Patienten mit AR darstellt [2]. Bei aller Unsicherheit der Analysen mit Modellvariablen lässt sich festhalten, dass sich eine AIT im deutschen Gesundheitssystem bereits nach 4–7 Jahren rechnet. Besonders relevant ist dabei die Langzeitwirkung [12, 23].

Trotz der evidenzbasierten, kausalen Therapiemöglichkeiten und der Kosteneffektivität erhielten 2021 in Deutschland nur ca. 6,1 % der diagnostizierten Patienten eine AIT (Antwort der Bundesregierung auf eine Kleine Anfrage der CDU/CSU-Fraktion).^2^ Eine Analyse der Daten von ca. 77 % aller bundesweit versicherten GKV-Patienten zeigt von 2021 auf 2022 einen deutlichen Rückgang initiierter AIT (je nach Allergen zwischen 13 und 31 %) [14]. Durch eine solche Unterversorgung können erhebliche Folgekosten für das Gesundheitswesen entstehen, z. B. durch ein mögliches Auftreten von AA [1, 23].

Die allergologische Versorgungslandschaft in Deutschland ist seit vielen Jahren ein regionaler Flickenteppich aus unterschiedlichen Regelungen [22]. In den meisten KV setzt sich die Ärztevergütung typischerweise mehrheitlich aus Regelleistungsvolumen (RLV), qualitätsgebundenem Zusatzvolumen (QZV) und förderungswürdigen Leistungen (FL) zusammen. Die Summe aus RLV und QZV multipliziert mit der Patientenzahl ergibt das Praxis-Gesamtbudget. Eine gute Praxisrentabilität wird erreicht, wenn die Summe aller erbrachten Leistungen in einem Quartal das Gesamtbudget ausschöpft oder höchstens unwesentlich überschreitet. Um Leistungen praxiswirtschaftlich zu beurteilen, sind die Honorierung und der Aufwand entscheidend.

Ziel dieser Arbeit ist die Darstellung und Bewertung der betriebswirtschaftlichen Aspekte einer AIT für den niedergelassenen HNO-Arzt und deren Folgewirkungen für den Therapieerfolg.

## Methoden

### Berechnung der Praxisrentabilität

Die Praxisrentabilität der Allergologie wurde anhand der allergologischen Leistungen für jede KV auf Basis des EBM und der KV-spezifischen Regelungen berechnet [20, 22]. Dabei wurden die Werte von 2022 zugrunde gelegt, da z. B. die DHPB von der Kassenärztlichen Bundesvereinigung (KBV) erst im Jahr 2024 veröffentlicht wurden (Orientierungspunktwert [OPW] 2022: 11,2662 Cent). Die Berechnung war für zwölf KV möglich (siehe elektronisches Zusatzmaterial online).

Es wurde eine optimale Adhärenz über drei Jahre bei einer perennialen AIT angenommen. Für die SCIT wurden drei Injektionen pro Quartal berechnet, außer im ersten Quartal, in dem eine Aufdosierung auf Erhaltungsdosis am ersten Behandlungstag angenommen wurde. Die Leistungen mit den entsprechenden Honorierungen sind in Tabelle S1 dargestellt.

Als Grundpauschale wurde die Gebührenordnungsposition (GOP) 09211 für Patienten im 6.–59. Lebensjahr gewählt. Dazu kamen die GOP 09220 (Zuschlag für die Hals-Nasen-Ohrenärztliche Grundversorgung) und die extrabudgetäre GOP 09222 (Zuschlag zur GOP 09220). Diese Pauschalen sind einmal im Behandlungsfall abrechenbar.

Die Berechnung fußt auf der Annahme, dass im ersten Quartal nach erfolgter Diagnostik direkt eine AIT beginnt. Dies ist möglich, wenn die Diagnostik zu Beginn des Quartals stattfindet, da AIT-Präparate im Regelfall innerhalb von vier Wochen lieferbar sind. Die GOP 30100 für die spezifische allergologische Anamnese wurde viermal im ersten Quartal (Q1) und einmal pro Quartal 5–12 abgerechnet.^3^ Als Vergleichsgröße dienten die DHPB aus den vier Honorarberichten der KBV von 2022: für Q1 der DHPB von Q1 im Jahr 2022, für Q2–Q4 der Durchschnittswert der DHPB aus Q2, Q3 und Q4 im Jahr 2022 und für Q5–Q12 der Durchschnittswert der DHPB aus den vier Quartalen 2022 [15–18].

Als Fallwert wurde der Durchschnitt aus den vier Quartalen 2022 aus den Angaben der einzelnen KV herangezogen (siehe Tabelle S2).

Die Berechnungen umfassen im Wesentlichen zwei Szenarien: 1.) Der Arzt bleibt im Rahmen seines Budgets und erhält somit eine Honorierung gemäß der EBM-Werte oder 2.) der Arzt bietet im Quartal Leistungen über das ihm genehmigte Budget hinaus an. Für die Überschreitung zum Fallwert wird ein auf 15 % reduziertes Honorar angenommen.

### Berechnung des Stundenhonorars und des Netto-Ertrags

Das Stundenhonorar errechnet sich aus der Vergütung und dem zeitlichen Aufwand. Hierzu wurden die Prüfzeiten aus Anhang 3 des EBM [20] zugrunde gelegt (siehe Tabelle S4).

Um den Netto-Ertrag zu ermitteln, wurden aus dem Stundenhonorar die Aufwendungen pro Stunde abgezogen. Diese wurden aus dem Jahresbericht 2022 des Zi-Praxis-Panels (ZiPP) berechnet. Der Median für die Gesamtaufwendungen (u. a. Kosten für Personal, Material, Labor, Miete und Nebenkosten für Praxisräume) je Inhaber liegt bei 173,1 Tsd. € jährlich. Bei einer durchschnittlichen Jahresarbeitszeit je Inhaber von 2151 h ergeben sich durchschnittliche Aufwendungen von 80,47 €/h [33]. Die Personalkosten haben dabei einen Anteil von 57 %, also 45,87 € [33].

## Ergebnisse

Die Berechnungen des Arzthonorars für die Mehrheit der KV sind ausführlich in den Tabellen S5–S40 angegeben. Um die Berechnung exemplarisch auszuführen, werden im Folgenden Rechnungen für die KV Bayern (KVB) dargestellt, der KV mit der größten Anzahl an niedergelassenen HNO-Ärzten [19].

### Vergütung der SCIT in Q1 in einer budgeteinhaltenden HNO-Praxis

Die Honorierung für HNO-Ärzte erfolgt entsprechend dem EBM, sofern das Budget eingehalten wird. In der KVB gelten für die Allergologie eine leistungsfallbezogene QZV und Zuschläge als FL. Für die Modellberechnung wurden im Q1 die Leistungen im oberen Bereich der Tab. 1 abgerechnet.

**Tab. 1:** Beispielhafte Berechnung des Honorars eines Hals-Nasen-Ohren(HNO)-Arztes für allergologische Diagnostik und Einleitung der subkutanen Immuntherapie (SCIT) im ersten Quartal (Q1) 2022 in der Kassenärztlichen Vereinigung Bayern (KVB). Im unteren Bereich sind die summierten Beträge für das Regelleistungsvolumen (RLV), das qualitätsgebundene Zusatzvolumen (QZV) (Diagnostik bzw. AIT) und extrabudgetäre Leistungen dargestellt. Zum Vergleich der Honorare ist auch das durchschnittliche Honorar pro Behandlungsfall (DHPB) aufgeführt

Anzahl	EBM-Ziffer	Leistung (SCIT)	EBM-Punkte	Wert (€)	Gesamtwert (€)
1	09211	Grundpauschale 6.–59. Lebensjahr	205	23,10	23,10
0	09220	Zuschlag für die HNO-Grundversorgung	27	3,04	0,00
0	09222	Zuschlag zu der GOP 09220	7	0,79	0,00
4	30100	Anamnese/Beratung	65	7,32	29,28
1	30111	Diagnostik	220	24,79	24,79
1	40351	Kostenpauschale Sachkosten	Pauschal	5,50	5,50
1	30120	Rhinomanometrischer Provokationstest	66	7,44	7,44
3	30130	Injektion Grund- und Fortsetzungsbehandlung	102	11,49	34,47
1	30131	Zuschlag für die 2. Injektion	80	9,01	9,01
*EBM (Einheitlicher Bewertungsmaßstab)-Vergütung RLV*					*23,10*
*EBM-Vergütung QZV Diagnostik*					*61,50*
*EBM-Vergütung QZV Hyposensibilisierung*					*43,49*
*EBM-Vergütung – Pauschalen, extrabudgetäre Leistungen und Sondervergütungen für evtl. förderungswürdige Leistungen (FL)*					*10,90*
*Gesamthonorar*					*138,99*
*DHPB in KVB in Q1 2022 *[15]					*57,54*

Die einzelnen Leistungen werden in den Zwischenrechnungen der EBM-Vergütung für RLV sowie QZV für Diagnostik und Hyposensibilisierung summiert (siehe Tab. 1).

In die RLV-Zwischenrechnung fallen die GOP 09211, 09220 und 09222. Es ist zu beachten, dass in dem Quartal, in dem die GOP 30120 abgerechnet wird, ein Ausschluss der Berechnungsfähigkeit der Pauschale für die fachärztliche Grundversorgung gilt [20].

Unter der QZV-Zwischenrechnung für Diagnostik werden die GOP 30100, 30111 und 30120 summiert.

Für die SCIT werden die GOP 30130 und 30131 (Zuschlag zur GOP 30130) addiert. Die 30131 wird bei einer zeitsparenden Aufdosierung in zwei Schritten auf die Erhaltungsdosis am ersten Behandlungstag abgerechnet, z. B. bei vereinzelten Allergoiden [9]. Bei längeren Aufdosierungsschemata würde die GOP 30130 häufiger zum Einsatz kommen [7, 8, 10].

In Bayern gelten die GOP 30130 und 30131 als FL und werden jeweils mit 1,35 € honoriert.^4^ Diese und die Kostenpauschalen zu u. a. der GOP 30111 werden extrabudgetär berechnet.

Für die SCIT ergibt sich in Q1 eine Gesamtsumme von 138,99 €, die den DHPB für HNO-Ärzte in Bayern, der im Q1 2022 bei 57,54 € lag, übersteigt (siehe Tab. 1).

### Vergütung der SCIT-Fortsetzungsbehandlung in einer budgeteinhaltenden HNO-Praxis

In der Modellberechnung für Q2–Q4 fließen bei einer SCIT die Grundpauschale, die fachärztlichen Zuschläge (GOP 09211, 09220 und 09222) und drei Injektionen (GOP 30130) ein. In den folgenden Quartalen wird zusätzlich die GOP 30100 abgerechnet. Der Vergleich mit den jeweiligen DHPB zeigt, dass eine SCIT in Bayern jedes Quartal überdurchschnittlich vergütet wird (siehe Tabelle S10).

Die Grundpauschale (GOP 09211) und die fachärztlichen Zuschläge für die HNO (GOP 09220 und 09221) tragen wesentlich zur Honorierung bei: Nach drei Jahren erhält ein HNO-Arzt allein für diese Positionen 319,33 € bei guter Patientenführung bzw. Adhärenz (siehe Tabelle S1).

### Das Stundenhonorar bei SCIT

Das Stundenhonorar ergibt sich aus dem Verhältnis der Honorierung zum zeitlichen Aufwand. Legt man die Prüfzeiten aus dem EBM der Zeitrechnung zugrunde, ergeben sich für alle Leistungen im Q1 50 min für die SCIT mit einem Stundenhonorar von 166,79 €/h.

In der Fortsetzungsbehandlung beträgt das Stundenhonorar 178,50 €/h bei jeweils 22 zur Verfügung stehenden Minuten in Q2–Q4 bzw. 161,71 €/h bei jeweils 27 min in Q5–Q12.

Um die Wirtschaftlichkeit zu beurteilen, werden dem Stundenhonorar die Aufwendungen von 80,47 €/h gegenübergestellt (berechnet aus dem Jahresbericht 2022 des ZiPP; siehe Methoden). Das ergibt bei einer SCIT einen Netto-Ertrag vor Abzügen von mindestens 81,24 €/h. Die Tabelle S42 zeigt eine Übersicht der Honorierung nach Abzug der Gesamtaufwendungen für alle zwölf untersuchten KV.

### Vergütung der SCIT in einer budgetüberschreitenden HNO-Praxis

Überschreitet der HNO-Arzt sein Budget, erhält er ein abgesenktes Honorar. In dieser Modell-Berechnung wird bei Leistungen oberhalb des Fallwerts der Überschuss nur mit 15 % des im EBM festgelegten Werts berechnet.

In Bayern ergeben sich in Q1 z. B. abgestaffelte Werte für das QZV für Diagnostik und Hyposensibilisierung von 36,27 € bzw. 21,83 € und somit ein angepasstes Gesamthonorar von 92,10 € für eine SCIT (siehe Tabelle S3).

In den folgenden Quartalen liegt die Vergütung bei jeweils 51,46 € in Q2–Q4 und 58,78 € in Q5–Q12 für die SCIT (siehe Tab. 2, S8 und S10). Trotz abgesenkter Honorare liegt die Vergütung bei einer SCIT meist über dem DHPB der KVB. Bei einer dreijährigen SCIT bei einem adhärenten Patienten beträgt das Gesamthonorar 716,67 €, also 32,92 € über dem Gesamt-DHPB (Tab. 2).

**Tab. 2:** Beispielhafte Berechnung des abgesenkten Honorars (gesamt und pro Quartal) eines Hals-Nasen-Ohren(HNO)-Arztes für allergologische Diagnostik und eine dreijährige subkutane Immuntherapie (SCIT) in der Kassenärztlichen Vereinigung Bayern (KVB) bei einem adhärenten Patienten. Bei der Vergütung wurde der Überschuss zum Fallwert nur mit 15 % berechnet. Zum Vergleich der Honorare ist auch das durchschnittliche Honorar pro Behandlungsfall (DHPB) dargestellt

Anzahl Quartale	Quartal	DHPB (€)	Honorar pro Quartal bei SCIT (€)
1	Q1	57,54	92,10
3	Q2–Q4	56,79	51,46
8	Q5–Q12	56,98	58,78
*12*	*Q1–Q12*	*683,75*	*716,67*

### Vergütung der SLIT

Für eine SLIT in der KVB beträgt das Honorar in einer budgeteinhaltenden HNO-Praxis in Q1 90,10 € und bei Überschreitung des Budgets 64,87 €. In der Fortsetzungsbehandlung beträgt die Vergütung für beide Szenarien 26,93 € in Q2–Q4 bzw. 34,25 € in Q5–Q12 und unterschreitet somit für elf Quartale den DBPH (siehe Tabelle S9). Bei einer SLIT bestünde die Möglichkeit, eine Lupenlaryngoskopie (GOP 09311) durchzuführen.

Das Stundenhonorar bei einer SLIT beträgt in Q1 138,62 €/h, in Q2–Q4 124,29 €/h und in Q5–Q12 114,17 €/h bei Prüfzeiten von jeweils 39, 13 bzw. 18 min. Nach Abzug der Aufwendungen ohne Berücksichtigung der Personalkosten ergibt sich ein Überschuss von mindestens 79,57 €. Die Tabelle S43 zeigt eine Übersicht der Honorierung nach Abzug der Gesamtaufwendungen für alle zwölf untersuchten KV.

### Deutschlandweite Honorierung von Diagnostik und dreijähriger AIT in HNO-Praxen

Die Berechnungen für die KV, in denen die RLV-, QZV- und FL-Zahlen in den KV-Portalen veröffentlicht wurden, sind in den Tabellen S5–S40 und in der Übersicht in Tab. 3 dargestellt.

**Tab. 3:** Beispielhafte Berechnung des abgesenkten Gesamthonorars eines Hals-Nasen-Ohren(HNO)-Arztes für allergologische Diagnostik (nur im ersten Quartal (Q1) der Therapie) und eine dreijährige allergenspezifische Immuntherapie (AIT) in zahlreichen Kassenärztlichen Vereinigungen (KV) bei einem adhärenten Patienten auf Basis von Zahlen von 2022. Die Differenz zum durchschnittlichen Honorar pro Behandlungsfall (DHPB) ist in Klammern dargestellt

	Gesamthonorar 3 Jahre (€)				
KV	DHPB	Diagnostik und SCIT		Diagnostik und SLIT	
		Budgeteinhaltende HNO-Praxis	Budgetüberschreitende HNO-Praxis	Budgeteinhaltende HNO-Praxis	Budgetüberschreitende HNO-Praxis
Baden-Württemberg	598,41	986,05 (387,64)	592,79 (−5,62)	452,85 (−145,56)	402,51 (−195,90)
Bayern	683,75	917,50 (233,75)	716,67 (32,92)	444,85 (−238,9)	419,62 (−264,13)
Berlin	622,67	1010,14 (387,47)	612,98 (−9,69)	598,83 (−23,84)	554,28 (−68,39)
Brandenburg	581,66	1052,58 (470,92)	610,64 (28,98)	444,87 (−136,79)	358,02 (−223,64)
Bremen	584,85	1057,82 (472,97)	622,21 (37,36)	444,87 (−139,98)	362,77 (−222,08)
Hamburg	696,72	981,46 (284,74)	825,35 (128,63)	451,87 (−244,85)	373,09 (−323,63)
Hessen	628,08	915,00 (286,92)	707,04 (78,96)	451,12 (−176,96)	427,37 (−200,71)
Niedersachsen	604,21	867,58 (263,37)	562,69 (−41,52)	444,87 (−159,34)	392,28 (−211,93)
Nordrhein	556,79	1054,10 (497,31)	590,14 (33,35)	477,87 (−78,92)	373,21 (−183,58)
Sachsen	627,38	927,58 (300,20)	815,22 (187,84)	444,87 (−182,51)	425,09 (−202,29)
Sachsen-Anhalt	592,18	867,58 (275,40)	704,24 (112,06)	444,87 (−147,31)	415,93 (−176,25)
Westfalen-Lippe	565,68	867,58 (301,90)	461,85 (−103,83)	444,87 (−120,81)	385,18 (−180,50)

In budgeteinhaltenden HNO-Praxen übertrifft das Honorar für Diagnostik und die dreijährige SCIT deutlich den DHPB in jeder KV und zeigt in budgetüberschreitenden HNO-Praxen, selbst bei abgesenkten Punktwerten, meist eine überdurchschnittliche Vergütung im Vergleich zum DHPB.

Unabhängig davon, ob das Budget eingehalten oder überschritten wird, erreicht eine HNO-Praxis bei allergologischer Diagnostik und SLIT in keiner der untersuchten KV das DHPB (siehe Tab. 3 und Zusatzmaterial).

### Vergleich der Honorierung bei SCIT und SLIT

Der Unterschied zwischen den beiden Applikationsformen besteht darin, dass die Ziffern 30130 und 30131 bei der SLIT nicht abgerechnet werden können. Bei 37 Injektionen wären das (ohne Überschreitungsquotierung) 422,65 € zusätzliche Einnahmen durch die SCIT (siehe Tabelle S1).

Nach drei Jahren beträgt in der KVB der Honorarunterschied zwischen SCIT und SLIT für budgetüberschreitende HNO-Praxen 297,05 €. Auch in den anderen KV fällt das Ergebnis zugunsten der SCIT aus (siehe Tabellen S5–S40).

Das Stundenhonorar beider Applikationen ist in der KVB vergleichbar, wenn man die unterschiedlichen Aufwendungen (bei SCIT mit, während bei SLIT ohne Personalkosten) mitberechnet, d. h., bei einer SCIT wird der zusätzliche personelle Aufwand durch 47,54 €/h kompensiert (siehe Abschnitte „Das Stundenhonorar bei SCIT“ und „Vergütung der SLIT“ und Tabellen S42–S44 für eine Übersicht der zwölf untersuchten KV).

## Diskussion

Allergische Erkrankungen stellen neben einer verringerten Lebensqualität für Betroffene auch ein massives gesundheitsökonomisches Problem für viele Volkswirtschaften dar [4].

Die AIT gilt als eine wirksame und sichere Behandlung, jedoch ist für den Therapieerfolg eine über mindestens drei Jahre hinweg anhaltende Adhärenz entscheidend, um einen krankheitsmodifizierenden Langzeit-Effekt zu erreichen [26]. Die mangelhafte Adhärenz gegenüber der AIT ist zudem mit hohen Kosten verbunden [4, 21].

RWE-Studien auf Basis von Verschreibungsdaten in Deutschland zeigen unabhängig von der Applikationsform eine sehr niedrige Adhärenz der AIT-Patienten. Sie ist niedriger als von ärztlicher Seite angenommen [27, 28, 32]. Aufgrund der Applikation der SCIT durch den Arzt ist ein Monitoring der Therapieadhärenz bei der SCIT einfacher als bei der SLIT [27].

Zur Steigerung der Adhärenz bei einer AIT werden Maßnahmen empfohlen, die teilweise von Ärzten und ihrem Praxisteam durchgeführt werden sollen [11, 27]. Sie sind zeitaufwendig und sollten praxiswirtschaftlich geprüft werden. In mehreren KV werden die allergologischen Leistungen als förderungswürdig erachtet, und einige haben auch eine Adhärenzziffer eingeführt, die z. B. bei erfolgreicher Beendigung der Therapie vergütet wird (siehe Tabelle S2).

Die AIT bietet die Möglichkeit, eine gute Arzt-Patienten-Bindung mit wirtschaftlichen Vorteilen aufzubauen. Das hier vorgestellte Modell berechnet die Vergütung in zwei Szenarien für die Diagnostik und eine dreijährige AIT. Dabei wurde in der Annahme, eine Leistung lohnt sich, wenn ihre Vergütung den DHPB erreicht oder überschreitet, eine quartalsweise erfolgende Betrachtung gewählt. Die allergologische Diagnostik und eine SCIT zeigen eine gute Praxisrentabilität in allen zwölf untersuchten KV bei Einhaltung des Budgets bzw. Honorierung nach EBM. In einem Szenario, in dem Leistungen, die über den jeweiligen KV-spezifischen Quartals-Fallwert von RLV bzw. QZV angeboten werden, lohnt sich die SCIT weiterhin in fast allen KV. Bei einer SLIT dagegen kann eine überdurchschnittliche Vergütung nur im ersten Quartal in Zusammenhang mit der Diagnostik erzielt werden (siehe Tabellen S5–S40). Für eine betriebswirtschaftlich wirksame Praxissteuerung soll der optimale Therapiemix der HNO-Praxis individuell ermittelt werden. Die SCIT ist gut honoriert und fällt daher für das Praxisbudget deutlich stärker ins Gewicht als die SLIT, die fast immer zu einer relevanten Unterdeckung führt [13].

Für den Therapieerfolg ist die Adhärenz entscheidend. Bei einer SCIT-Fortsetzungsbehandlung wird das jeweilige DHPB eines Quartals meist übertroffen, auch in der Berechnung mit abgesenktem Honorar (siehe Tabellen S5–S40). Allein die HNO-Grundpauschale und ihre Zuschläge werden KV-übergreifend mit 319,33 € für eine dreijährige Therapie vergütet. Das bedeutet, dass eine gut organisierte allergologisch tätige HNO-Praxis, die durch optimale Abläufe und Logistik einen geringen zeitlichen Aufwand bei der Versorgung der regelmäßig wiederkehrenden AIT-Patienten hat, ein durchaus positives Aufwand-Ertrag-Verhältnis mit der Allergologie erreichen kann.

Auf Basis der Prüfzeiten im EBM werden die Diagnostik und eine dreijährige Therapie für 5 h und 32 min (SCIT) bzw. für 3 h und 42 min (SLIT) honoriert. Das daraus berechnete Stundenhonorar beträgt je nach Quartal mindestens 161,71 €/h (SCIT) bzw. 114,17 €/h (SLIT). Nach Abzug der Aufwendungen (ohne Personalkosten für SLIT) sind die Netto-Erträge für beide Applikationsformen vergleichbar.

Die für die GOP hinterlegten Prüfzeiten, mit denen als zeitliche Grundlage die Stundenhonorare berechnet wurden, scheinen für den Praxisalltag eher lang (z. B. 18 min für den einmalig im Quartal stattfindenden Besuch eines SLIT-Patienten), sodass der Erlös pro Stunde bei SCIT und SLIT praktisch höher sein dürfte.

Zur Vervollständigung wird hier auch der nur symptomatisch behandelte Patient, der keinerlei AIT erhält, mit angefügt: Dieser erbringt neben der Grundpauschale, den Zusatzpauschalen und ggf. FL in den dem Diagnostikquartal folgenden Quartalen analog zu einem SLIT-Patienten keine weiteren Zusatzeinnahmen, z. B. durch die Injektionen einer SCIT. Zusätzlich gilt es hier noch zwischen ganzjähriger allergischer Symptomatik, bei der der Patient ggf. in allen vier Quartalen im Jahr in die Praxis kommt und damit wie bei einer SLIT vergütet wird, und saisonaler allergischer Symptomatik zu unterscheiden, bei der der Patient unter Umständen nur in einem oder zwei Quartalen den HNO-Arzt aufsucht. Im betriebswirtschaftlich ungünstigsten Szenario für den Arzt kommt der Patient nach dem Diagnostikquartal nicht mehr in die Praxis und führt eine symptomatische Therapie ausschließlich selbstständig mit nicht verschreibungspflichtigen Präparaten ohne weiteren Arztkontakt durch.

Dies ist nach Kenntnis der Autoren die erste Übersichtsarbeit, die das Honorar von HNO-Ärzten für die Allergologie in zahlreichen KV berechnet. Das Modell konnte zeigen, dass sich der Aufwand einer AIT – auch über den empfohlenen Zeitraum von drei Jahren – durchaus in der Praxisrealität lohnt: bei beiden Applikationsformen durch das regelmäßige Erscheinen des Patienten in der Praxis über drei Jahre bzw. zwölf Quartale und insbesondere bei einer SCIT durch zusätzliche Einnahmen, auch unter Berücksichtigung des erhöhten Personalaufwandes.

Dies unterstreicht explizit noch einmal den Wert der Therapieadhärenz nicht nur aus medizinischen, sondern auch aus betriebswirtschaftlichen Gründen. Die Autoren hoffen, damit einen weiteren Grund zu liefern, die Adhärenz zu optimieren und grundsätzlich der allergologischen Unterversorgung entgegenzuwirken.

Diese Analyse unterliegt gewissen Einschränkungen. Es wird darauf hingewiesen, dass diese Berechnungen auf bestimmten Annahmen, Modellen und Daten beruhen (siehe Methoden), die möglicherweise mit Unsicherheiten behaftet sind und oft nicht im Alltag jeder Praxis bzw. KV in dieser Form umgesetzt werden (können). Die Autoren empfehlen daher, diese Ergebnisse mit angemessener Vorsicht zu interpretieren und ggf. zusätzliche Überprüfungen oder Validierungen durchzuführen, um ihre Gültigkeit zu bestätigen. Auch wurden nur die allergologischen Leistungen betrachtet. Die Verteilung und demnach die Überschreitung des Budgets hängt aber auch von Faktoren wie dem Verordnungsverhalten der Fachgruppe und der Gesamtheit der Leistungen ab. Letzteres wirkt sich in unserer Modellberechnung der abgesenkten Honorare insbesondere auf die SCIT aus, da diese z. B. die RLV-Obergrenze eher überschreitet. Dennoch bleibt die Überzeugung, dass die Analyse eine solide Grundlage für das ökonomische Verständnis der AIT und die Vorhersage in realen Szenarien bietet.

## Fazit für die Praxis

- Diese Modellberechnungen zeigen, dass die allergologische Diagnostik und die AIT betriebswirtschaftlich vorteilhaft für HNO-Praxen sind.
- Für eine gute Praxisrentabilität ist hierbei eine möglichst hohe Adhärenz der Patienten über drei Jahre Therapie entscheidend.
- Die Fortsetzungsbehandlung zeigt ein positives Aufwand-Ertrag-Verhältnis und unterstützt somit die Forderung nach einer therapietreuen dreijährigen AIT, die bisher die einzige kausale Therapieoption darstellt.

## Supplementary Information

KV-spezifische Berechnung des Honorars von Diagnostik und dreijähriger AIT sowohl in budgeteinhaltenden als auch in budgetüberschreitenden HNO-Praxen sowie Vergleich der Stunden-Honorierung bei SCIT und SLIT in einer budgeteinhaltenden Praxis

## Data Availability

Alle Daten, die die Ergebnisse dieser wissenschaftlichen Publikation stützen, sind innerhalb des vorliegenden Artikels und/oder in den ergänzenden Dateien/Materialien bzw. offen verfügbar (links in den jeweiligen Quellenverzeichnisse enthalten).

## Abkürzungen

AA: Allergisches Asthma
AIT: Allergenspezifische Immuntherapie; „allergen-specific immunotherapy“
AR: Allergische Rhinitis
DHPB: Durchschnittliches Honorar pro Behandlungsfall
EBM: Einheitlicher Bewertungsmaßstab
ENT: „Ear, nose, and throat physician“
FL: Förderungswürdige Leistungen
GOP: Gebührenordnungsposition
HNO: Hals-Nasen-Ohren-Arzt
KV: Kassenärztliche Vereinigung
KBV: Kassenärztliche Bundesvereinigung
KVB: Kassenärztliche Vereinigung Bayern
OPW: Orientierungspunktwert
Q1: Quartal 1; erstes Quartal einer AIT
Q2–4: Quartale 2 bis 4; zweites bis viertes Quartal einer AIT
Q5–12: Quartal 5 bis 12; fünftes bis zwölftes Quartal einer AIT
QZV: Qualitätsgebundenes Zusatzvolumen
RLV: Regelleistungsvolumen
RWE: „Real-world evidence“
SCIT: Subkutane Immuntherapie
SLIT: Sublinguale Immuntherapie
ZiPP: Zi-Praxis-Panels (Praxis-Panel des Zentralinstituts für die kassenärztliche Versorgung in der Bundesrepublik Deutschland)

## Footnotes

1 Wir möchten hervorheben, dass wenn im Folgenden das generische Maskulinum verwendet wird, immer Frauen, Männer sowie andere Geschlechtsidentitäten gemeint sind.

2 BT-Drucks. 20/3180, S. 4.

3 Zu beachten gilt, dass die GOP 30100 den obligatorischen persönlichen Arzt-Patienten-Kontakt vorsieht und je vollendete fünf Minuten höchstens viermal im Krankheitsfall berechnungsfähig ist. Ein Krankheitsfall umfasst das aktuelle sowie die drei nachfolgenden Kalendervierteljahre.

4 Die Abrechnung der Nummern setzt voraus, dass die Praxis ein geeignetes Verfahren einsetzt, um Patienten, die sich einer SCIT unterziehen, an die regelmäßige Wahrnehmung ihrer Behandlungstermine zu erinnern.
