# Supplementary material for: Is AIT worth your while? Economic evaluation of allergen-specific immunotherapy (AIT) for otorhinolaryngologists in private practice
Source: HNO. 2025 Feb 3;73(4):225–32. [Article in German] doi: 10.1007/s00106-024-01542-8 (PMC11926047; doi:10.1007/s00106-024-01542-8)
Supplement: Supplementary file 1 — KV-spezifische Berechnung des Honorars von Diagnostik und dreijähriger AIT sowohl in budgeteinhaltenden als auch in budgetüberschreitenden HNO-Praxen sowie Vergleich der Stunden-Honorierung bei SCIT und SLIT in einer budgeteinhaltenden Praxis [file 106_2024_1542_MOESM1_ESM.pdf]

KASSENÄRZTLICHE VEREINIGUNGS(KV)-SPEZIFISCHE BERECHNUNG DES HONORARS VON DIAGNOSTIK UND DREIJÄHRIGER AIT SOWOHL IN BUDGETEINHALTENDEN ALS AUCH IN BUDGETÜBERSCHREITENDEN HNO-PRAXEN SOWIE VERGLEICH DER STUNDEN-HONORIERUNG BEI SUBKUTANER IMMUNTHERAPIE (SCIT) UND SUBLINGUALER IMMUNTHERAPIE (SLIT) IN EINER BUDGETEINHALTENDEN PRAXIS

METHODEN

Regelleistungsvolumen (RLV), Qualifikationsgebundenes Zusatzvolumen (QZV) und förderungswürdige Leistungen (FL)

QZVs können je nach KV verschieden berechnet werden: RLV-fallbezogen, QZV-leistungsfallbezogen oder arztbezogen.

RLV-fallbezogene QZV gelten in den KV in Baden-Württemberg, Hessen, Mecklenburg-Vorpommern und Westfalen-Lippe (KVBaWü, KVH, KVMV, KVWL). Die Berechnung findet auf Basis aller RLV-Fälle einer Praxis statt (egal welche Behandlung).

Eine leistungsfallbezogene QZV, d.h. auf Basis der Anzahl QZV-relevanter Abrechnungsziffern, gilt für HNO-Ärzte in den KV in Bayern, Nordrhein, Sachsen und Sachsen-Anhalt (KVB, KVNo, KVS, KVSA).

In der KV in Berlin, Niedersachsen, Rheinland-Pfalz, Thüringen, Schleswig-Holstein und Saarland (KVBerlin, KVN, KVRP, KVT, KVSH, KVSaarland) gilt ein arztbezogenes QZV, dabei wird das zur Verfügung stehende Gesamtvergütungsvolumen der jeweiligen QZV-Leistung durch die Anzahl der Ärzte geteilt, die einen Anspruch auf dieses QZV haben, d.h. unabhängig von der Anzahl der erbrachten Leistungen erhält jeder Arzt das gleiche QZV-Volumen.

In den KV in Brandenburg, Bremen und Hamburg (KVBB, KVHB und KVHH) werden keine Leistungen über QZVs vergütet.

Förderungswürdige Leistungen (FL) werden außerhalb der morbiditätsbedingten Gesamtvergütung (MGV) gesondert vergütet – ohne Mengenbegrenzung für Leistungen, die Krankenkassen für besonders förderungswürdig erachten. Derzeit wird die AIT in 12 von 17 KV-Bereichen gefördert (1).

Berechnung der Praxisrentabilität

Die Praxisrentabilität der Allergologie wurde anhand der allergologischen Leistungen für jede KV auf Basis des einheitlichen Bewertungsmaßstabes (EBM) und der KV-spezifischen Regelungen berechnet (1,2). Dabei wurden die Werte von 2022 genommen, da z.B. die durchschnittlichen Honorare pro Behandlungsfall (DHPB) von der KBV erst in 2024 veröffentlicht werden. Der Orientierungspunktwert (OPW) betrug in 2022 11,2662 Cent je Punkt (siehe Methoden).

Eine Berechnung war nur für 12 von 17 KV möglich, da die Zahlen in den anderen 5 KV individuell berechnet werden. In Mecklenburg-Vorpommern z.B. gilt die Hyposensibilisierungsbehandlung (Gebührenordnungsposition (GOP) 30130, 30131) als FL und wird individuell berechnet. Individualbudgets gelten in Rheinland-Pfalz sowie in Thüringen, Praxisbudgets in Saarland und arztindividuelle Punktzahlvolumina in Schleswig-Holstein (3–6).

Es wurde eine optimale Adhärenz über drei Jahre angenommen. Für die SCIT wurden drei Injektionen pro Quartal berechnet, außer im ersten Quartal, in dem eine Aufdosierung auf Erhaltungsdosis am ersten Behandlungstag angenommen wurde. Die angenommenen Leistungen und die entsprechende Honorierung sind in Tabelle S1 dargestellt.

EBM 2022		SCIT				SLIT			
GOP	Leistung	Punkte	Anzahl (N)	Wert (€)	Gesamt-Wert (€)	Punkte	Anzahl (N)	Wert (€)	Gesamt-Wert (€)
09211	Grundpauschale 6.-59. Lebensjahr	205	12	23,10	277,20	205	12	23,10	277,20
09220	Zuschlag für die HNO-Grundversorgung	27	11	3,04	33,44	27	11	3,04	33,44
09222	Zuschlag zu der Gebührenordnungs position 09220	7	11	0,79	8,69	7	11	0,79	8,69
30100	Anamnese	65	12	7,32	87,84	65	12	7,32	87,84
30111	Pricktestung	220	1	24,79	24,79	220	1	24,79	24,79
40351	Kostenpauschale		1	5,50	5,50			5,50	5,50
30120	Rhinom. Provokationstest	66	1	7,44	7,44	66	1	7,44	7,44
30130	Hypo-sensibilisierung	102	36	11,49	413,64				
30131	2. Hyposens. Injektion am Tag 1	80	1	9,01	9,01				
Summe					867,55				444,90

Tab. S1: EBM-Honorierung der allergologischen Diagnostik und einer AIT (SCIT und SLIT) über drei Jahre für das Jahr 2022 mit einem OPW von 0,112662 € unter Berücksichtigung der Grundpauschalen und ihrer Zuschläge. Förderungswürdige Leistungen wurden nicht berücksichtigt. Es ist zu beachten, dass in dem Quartal, in dem die GOP 30120 abgerechnet wird, ein Ausschluss der Berechnungsfähigkeit der Pauschale für die fachärztliche Grundversorgung gilt (2).

Berechnung der Praxisrentabilität in einer HNO-Praxis, die das Budget überschreitet

Bei der Annahme, der Arzt würde im Quartal Leistungen über das ihm genehmigte Budget hinaus anbieten, wurde für die Überschreitung zum Fallwert ein auf 15 % reduziertes Honorar angenommen. Als Fallwert für die RLV bzw. QZV wurde der Durchschnitt der Fallwerte aus den vier Quartalen 2022 berechnet gemäß der Angaben der einzelnen KV (siehe Tabelle S2).

Kennzahl	Zusatzinformation	Quartal 2022	Fallwert (€)
KVBaWü (7)			
RLV		1	33,15
		2	35,66
		3	29,69
		4	31,75
FL	Zuschlag zur GOP 30130 EBM	1-4	3,00
	Zuschlag zur GOP 30131 EBM		2,50
	Zuschlag zur GOP 30111 EBM		8,00
KVB (8,9)			
RLV (ALK6-59)		1-4	27,11
QZV	Diagnostik (30100, 30110, 30111, 30120, 30121, 30122, 30123)	1-4	31,82
	Hyposensibilisierung (30130, 30131)	1-4	18,01
FL	Zuschlag zu jeder abgerechneten und anerkannten GOP 30130 (Abrechnungsnummer 97142) bzw. 30131 (Abrechnungsnummer 97142A)	1-4	1,35

KV Berlin (10,11)			
BEV		1-2	34,33
		3	28,87
		4	31,19
FL	SCIT (GOP 30130) und SLIT (SNR 30130T)	1-4	0,03 pro Punkt (= 3,06 € SCIT) 10,33 (SLIT)
	Die kontinuierliche Hyposensibilisierungstherapie über 3 Jahre wird mit Ablauf des Behandlungszeitraums einmalig mit einem Zuschlag von 30 € gefördert: SNR 91130		30,00
KV Brandenburg (12)			
RLV		1-2	28,29
		3-4	27,29
FL	GOP 30130, 30131 -	1-4	5,00
KVHB (13,14)			
RLV		1	34,27
		2	34,62
		3	20,89
		4	23,87
FL	Zuschlag Hyposensibilisierung (GOP 99054, automatisch von KV zugesetzt)	1-2	3,34
		3-4	3,38
	Zuschlag für erfolgreiche Beendigung subkutane Therapie (GOP 99055)	1-2	70,00
		3-4	70,89
KVHH (15,16)			
Garantie-quoten		1	77 %
		2	87 %
		3	79 %
		4	84 %
FL	Zuschlag für Hyposensibilisierungsbehandlungen (GOP 30130, 30131) nach 3 Jahren Behandlungsdauer einmal je Patienten (GOP 98000).		100
KVH (17–19)			
RLV		1	49,83
		2	50,51
		3	50,37
		4	50,04
QZV	GOP 30100, 30110, 30111, 30120-30123, 30130, 30131	1-4	
FL	GOP 30111, 30120, 30130, 30131	1-4	11,2% auf dem EBM-Wert 2018
KVN (20)			
RLV		1	24,47
		2	30,33
		3	22,84
		4	23,66
QZV	Diagnostik (30100, 30110, 30111, 30120, 30121, 30122, 30123	1	10,69
		2	13,80
		3	4,23

		4	5,80
	Hyposensibilisierung (30130, 30131)	1	13,86
		2	15,01
		3	5,08
		4	8,04
KVNo (21)			
RLV		1	26,01
		2	24,22
		3	26,53
		4	25,74
FL	Allergologie (30110, 30111, 30120, 30121, 30122, 30123)	1	17,94
		2	12,39
		3	16,01
		4	19,67
	Hyposensibilisierung (30130, 30131)	1	3,75
		2	4,35
		3	4,16
		4	3,91
KVS (22,23)			
RLV		1	25,88
		2	25,49
		3	25,68
		4	26,45
QZV	Diagnostik (30110, 30111, 30120, 3011, 30122, 30123)	1	40,61
		2	40,90
		3	40,75
		4	41,97
	Hyposensibilisierung (30130, 30131)	1	26,31
		2	25,60
		3	25,96
		4	26,74
FL	Der erste Arzt-Patienten-Kontakt im zweiten Krankheitsfall (2. Behandlungsjahr) (Abrechnungsziffer 99675A)		20,00
	Der erste Arzt-Patienten-Kontakt im dritten Krankheitsfall (3. Behandlungsjahr) (Abrechnungsziffer 99675B)		40,00
KVSA (24)			
RLV		1	25,71
		2	28,29
		3	27,75
		4	26,179
QZV		1	26,76
		2	30,10
		3	26,60
		4	26,35
		1	24,06
		2	21,17

		3	20,71
		4	22,27
KVWL (25)			
RLV (ALK6-59)		1	33,07
		2	31,24
		3	29,06
		4	32,00

Tabelle S2: Überblick der RLV- und QZV-Fallwerte sowie der förderungswürdigen Leistungen in den untersuchten KV für das Jahr 2022.
ALK: Altersklasse; BEV: Basis-EURO-Volumen; SNR: Symbolnummer.

Im Falle der Angaben von RLV- und QZV-Fallwerten wurden die jeweiligen Leistungen zum jeweiligen Fallwert angepasst (siehe z.B. die Berechnung für die KVB in Tabelle S3). Wurde nur der RLV-Fallwert angegeben, wurde dieser als ausschlaggebender Referenzwert verwendet (siehe z.B. Tabelle S5).

**Leistungsart**	**Fallwert (€)**	**EBM-Honorar SCIT (€)**	**15% der Über-schreitung (€)**	**Abgesenktes Honorar SCIT (€)**
RLV	27,11	23,10	0,00	23,10
QZV Diagnostik	31,82	61,50	4,45	36,27
QZV Hyposensibilisierung	18,01	43,49	3,82	21,83
Pauschalen, Extrabudgetäre Leistungen und Sondervergütungen für evtl. FL		10,90	0,00	10,90
**Honorar**		**138,99**		**92,10**
**DHPB in KVB in Q1 2022 laut Honorarbericht der KBV** (26)	**57,54**			

Tabelle S3: Beispielhafte Berechnung des abgesenkten Honorars eines HNO-Arztes für allergologische Diagnostik und Einleitung der SCIT im Quartal 1 2022 in der KVB. Dargestellt sind die summierten Beträge für RLV, QZV (Diagnostik bzw. SCIT), und extrabudgetäre Leistungen. wobei die Überschreitung aus dem EBM-Honorar (siehe Tabelle 1 (2)) im Vergleich zum Fallwert nur mit 15% berechnet wurde. Als Fallwert diente der durchschnittliche Fallwert für die Quartale 1-4 2022.

Berechnung des zeitlichen Aufwands, des Stundenhonorars und des Netto-Ertrags einer AIT

Die Berechnung des zeitlichen Aufwands einer AIT basiert auf den Angaben der Prüfzeiten aus dem Anhang 3 des EBM (2)

EBM 2022		SCIT			SLIT		
GOP	Legende	Prüfzeit (Min)	N	Gesamt-Zeit (Min)	Prüfzeit (Min)	N	Gesamt-Zeit (Min)
09211	Grundpauschale 6.-59. Lebensjahr	13	12	156	13	12	156
09220	Zuschlag für die Hals-Nasen-Ohrenärztliche Grundversorgung(in MVG)	0	11	0	0	11	0
09222	Zuschlag zu der GOP 09220 (Extrabudgetär)	0	11	0	0	11	0
30100	Anamnese	5	12	60	5	12	60
30111	Pricktestung	3	1	3	3	1	3
40351	Kosten-pauschale						
30120	Rhinom. Provokationstest	3	1	3	3	1	3
30130	Hypo-sensibilisierung	3	36	108			
30131	2. Hyposens. am Tag 1	2	1	2			
Summe				332			222

Tab. S4 Prüfzeiten in Minuten für die Grundpauschale, ihre Zuschläge, die allergologische Diagnostik und eine AIT (SCIT und SLIT) über drei Jahre gemäß Anhang 3 des EBM (2).

Das Stundenhonorar errechnet sich aus der Vergütung und dem zeitlichen Aufwand. Um den Netto-Ertrag zu ermitteln, wurden aus dem Stundenhonorar die Aufwendungen pro Stunde abgezogen. Diese wurden aus dem Jahresbericht 2022 des Zi-Praxis-Panels (ZiPP) berechnet. Der Median für die Gesamtaufwendungen (u. a. Kosten für Personal, Material, Labor, Miete und Nebenkosten für Praxisräume) je Inhaber liegt bei 173,1 Tsd. € jährlich. Bei einer durchschnittlichen Jahresarbeitszeit je Inhaber von 2.151 Stunden ergeben sich durchschnittliche Aufwendungen von 80,47 €/h (27). Die Personalkosten haben dabei einen Anteil von 57 %, also 45,87 € (27).

ERGEBNISSE

Baden-Württemberg

Vergütung der AIT in einer budgetüberschreitenden HNO-Praxis

**Pos.**	**EBM Ziffer**	**Leistung (SCIT)**	**Leistung (€)**	**N**	**Gesamt (€)**	**N**	**Gesamt (€)**	**N**	**Gesamt (€)**
				**Q1**		**Q2-4**		**Q5-12**	
1	09211	Grundpauschale 6.-59. Lebensjahr	23,10	1	23,10	1	23,10	1	23,10
2	09220	Zuschlag für die HNO Grundversorgung	3,04	0	0,00	1	3,04	1	3,04
3	09222	Zuschlag zu der GOP 09220	0,79	0	0,00	1	0,79	1	0,79
4	30100	Anamnese/Beratung	7,32	4	29,28	0	0,00	1	7,32
5	30111	Diagnostik	24,79	1	24,79	0	0,00	0	0,00
6	40351	Kostenpauschale Sachkosten	5,50	1	5,50	0	0,00	0	0,00
7	30120	Rhinomanometr. Provokationstest	7,44	1	7,44	0	0,00	0	0,00
8	30130	Injektion Grund- und Fortsetzungs-behandlung	11,49	3	34,47	3	34,47	3	34,47
9	30131	Zuschlag für die 2. Injektion	9,01	1	9,01	0	0,00	0	0,00
**EBM-Vergütung RLV (Summe Pos 1,2,4,5,7,8,9)**					**128,08**		**60,61**		**67,93**
**Pauschalen und extrabudgetäre Leistungen (Summe Pos. 3,6 und evtl. FL)**					**25,00**		**9,79**		**9,79**
Durchschnittlicher RLV-Fallwert					32,56		32,56		32,56
					**Gesamt (€)**		**Gesamt (€)**		**Gesamt (€)**
Abgesenkte Vergütung (AV) RLV - Überschreitung zum durchschnittlichen Fallwert (DF) mit 15% berechnet					46,89		36,77		37,87
Pauschalen und extrabudgetäre Leistungen (Summe Pos. 3,6 und evtl. FL)					25,00		9,79		9,79
**Gesamthonorar**					**71,89**		**46,56**		**47,65**
DHPB in Q1, Q2-Q4 bzw. Q1-Q4 2022 (26,28–30)					50,35		49,70		49,87

Tabelle S5: Beispielhafte Berechnung des Honorars eines HNO-Arztes für allergologische Diagnostik und SCIT in 2022 in der KVBaWü. Der DF wurde zur Berechnung der abgesenkten RLV-Vergütung verwendet. Zum Vergleich der Honorare ist auch das DHPB aufgeführt.

Pos.	EBM Ziffer	Leistung (SLIT)	Leistung (€)	N	Gesamt (€)	N	Gesamt (€)	N	Gesamt (€)
				Q1		Q2-4		Q5-12	
1	09211	Grundpauschale 6.-59. Lebensjahr	23,10	1	23,10	1	23,10	1	23,10
2	09220	Zuschlag für die HNO Grundversorgung	3,04	0	0,00	1	3,04	1	3,04
3	09222	Zuschlag zu der GOP 09220	0,79	0	0,00	1	0,79	1	0,79
4	30100	Anamnese/Beratung	7,32	4	29,28	0	0,00	1	7,32
5	30111	Diagnostik	24,79	1	24,79	0	0,00	0	0,00
6	40351	Kostenpauschale Sachkosten	5,50	1	5,50	0	0,00	0	0,00
7	30120	Rhinomanometr. Provokationstest	7,44	1	7,44	0	0,00	0	0,00
**EBM-Vergütung RLV (Summe Pos 1,2,4,5,7)**					**84,60**		**26,14**		**33,46**
**Pauschalen und extrabudgetäre Leistungen (Summe Pos. 3,6 und evtl. FL)**					**13,50**		**0,79**		**0,79**
Durchschnittlicher RLV-Fallwert					32,56		32,56		32,56
					**Gesamt (€)**		**Gesamt (€)**		**Gesamt (€)**
Abgesenkte Vergütung (AV) RLV - Überschreitung zum durchschnittlichen Fallwert (DF) mit 15% berechnet					40,37		26,14		32,69
Pauschalen und extrabudgetäre Leistungen (Summe Pos. 3,6 und evtl. FL)					13,50		0,79		0,79
**Gesamthonorar**					**53,87**		**26,93**		**33,48**
DHPB in Q1, Q2-Q4 bzw. Q1-Q4 2022 (26,28–30)					50,35		49,70		49,87

Tabelle S6: Beispielhafte Berechnung des Honorars eines HNO-Arztes für allergologische Diagnostik und SLIT in 2022 in der KVBaWü. Der DF wurde zur Berechnung der abgesenkten RLV-Vergütung verwendet. Zum Vergleich der Honorare ist auch das DHPB aufgeführt.

Honorar-Übersicht von Diagnostik und dreijähriger AIT in der KVBaWü

Anzahl Quartale	Quartale	DHPB (€)	Vergütung in einer budgeteinhaltenden Praxis (€)		Vergütung in einer budgetüberschreitenden Praxis (€)	
			Diagnostik (nur in Q1) und SCIT	Diagnostik (nur in Q1) und SLIT	Diagnostik (nur in Q1) und SCIT	Diagnostik (nur in Q1) und SLIT
1	Q1	50,35	153,08	98,10	71,89	53,87
3	Q2-4	49,70	70,40	26,93	46,56	26,93
8	Q5-12	49,87	77,72	34,25	47,65	33,48
**12**	**Q1-12**	**598,41**	**986,05**	**452,85**	**592,79**	**402,51**

Tabelle S7: Darstellung des maximal erhältlichen und des abgesenkten Honorars (gesamt und pro Quartal) eines HNO-Arztes für allergologische Diagnostik und eine dreijährige AIT in der KVBaWü bei einem adhärenten Patienten. Zum Vergleich der Honorare ist auch das Quartals- und Gesamt-DHPB dargestellt.

Bayern

Vergütung der AIT in einer budgetüberschreitenden HNO-Praxis

**Pos.**	**EBM Ziffer**	**Leistung (SCIT)**	**Leistung (€)**	**N**	**Gesamt (€)**	**N**	**Gesamt (€)**	**N**	**Gesamt (€)**
				**Q1**		**Q2-4**		**Q5-12**	
1	09211	Grundpauschale 6.-59. Lebensjahr	23,10	1	23,10	1	23,10	1	23,10
2	09220	Zuschlag für die HNO Grundversorgung	3,04	0	0,00	1	3,04	1	3,04
3	09222	Zuschlag zu der GOP 09220	0,79	0	0,00	1	0,79	1	0,79
4	30100	Anamnese/Beratung	7,32	4	29,28	0	0,00	1	7,32
5	30111	Diagnostik	24,79	1	24,79	0	0,00	0	0,00
6	40351	Kostenpauschale Sachkosten	5,50	1	5,50	0	0,00	0	0,00
7	30120	Rhinomanometr. Provokationstest	7,44	1	7,44	0	0,00	0	0,00
8	30130	Injektion Grund- und Fortsetzungs-behandlung	11,49	3	34,47	3	34,47	3	34,47
9	30131	Zuschlag für die 2. Injektion	9,01	1	9,01	0	0,00	0	0,00
**EBM-Vergütung RLV (Summe Pos. 1,2)**					**23,10**		**26,14**		**26,14**
**EBM-Vergütung QZV Diagnostik (Summe Pos. 4,5,7)**					**61,50**		**0,00**		**7,32**
**EBM-Vergütung QZV Hyposensibilisierung (Summe Pos. 8,9)**					**43,49**		**34,47**		**34,47**
**Pauschalen und extrabudgetäre Leistungen (Summe Pos. 3,6 und evtl. FL)**					**10,90**		**4,84**		**4,84**
Durchschnittlicher RLV-Fallwert					27,11		27,11		27,11
Durchschnittlicher QZV Diagnostik-Fallwert					31,82		31,82		31,82
Durchschnittlicher QZV Hyposens.-Fallwert					18,01		18,01		18,01
					**Gesamt (€)**		**Gesamt (€)**		**Gesamt (€)**
AV RLV - Überschreitung zum DF mit 15% berechnet					23,10		26,14		26,14
AV QZV Diagnostik - Überschreitung zum DF mit 15% berechnet					36,27		0,00		7,32
AV QZV Hyposens. - Überschreitung zum DF mit 15% berechnet					21,83		20,48		20,48
Pauschalen und extrabudgetäre Leistungen (Summe Pos. 3,6 und evtl. FL)					10,90		4,84		4,84
**Gesamthonorar**					**92,10**		**51,46**		**58,78**
DHPB in Q1, Q2-Q4 bzw. Q1-Q4 2022 (26,28–30)					57,54		56,79		56,98

Tabelle S8: Beispielhafte Berechnung des Honorars eines HNO-Arztes für allergologische Diagnostik und SCIT in 2022 in der KVB. Der DF wurde zur Berechnung der abgesenkten RLV-Vergütung verwendet. Zum Vergleich der Honorare ist auch das DHPB aufgeführt.

Pos.	EBM Ziffer	Leistung (SLIT)	Leistung (€)	N	Gesamt (€)	N	Gesamt (€)	N	Gesamt (€)
				Q1		Q2-4		Q5-12	
1	09211	Grundpauschale 6.-59. Lebensjahr	23,10	1	23,10	1	23,10	1	23,10
2	09220	Zuschlag für die HNO Grundversorgung	3,04	0	0,00	1	3,04	1	3,04
3	09222	Zuschlag zu der GOP 09220	0,79	0	0,00	1	0,79	1	0,79
4	30100	Anamnese/Beratung	7,32	4	29,28	0	0,00	1	7,32
5	30111	Diagnostik	24,79	1	24,79	0	0,00	0	0,00
6	40351	Kostenpauschale Sachkosten	5,50	1	5,50	0	0,00	0	0,00
7	30120	Rhinomanometr. Provokationstest	7,44	1	7,44	0	0,00	0	0,00
**EBM-Vergütung RLV (Summe Pos. 1,2)**					**23,10**		**26,14**		**26,14**
**EBM-Vergütung QZV Diagnostik (Summe Pos. 4,5,7)**					**61,50**		**0,00**		**7,32**
**Pauschalen und extrabudgetäre Leistungen (Summe Pos. 3,6 und evtl. FL)**					**5,50**		**0,79**		**0,79**
Durchschnittlicher RLV-Fallwert					27,11		27,11		27,11
Durchschnittlicher QZV Diagnostik-Fallwert					31,82		31,82		31,82
					**Gesamt (€)**		**Gesamt (€)**		**Gesamt (€)**
AV RLV - Überschreitung zum DF mit 15% berechnet					23,10		26,14		26,14
AV QZV Diagnostik - Überschreitung zum DF mit 15% berechnet					36,27		0,00		7,32
Pauschalen und extrabudgetäre Leistungen (Summe Pos. 3,6 und evtl. FL)					5,50		0,79		0,79
**Gesamthonorar**					**64,87**		**26,93**		**34,25**
DHPB in Q1, Q2-Q4 bzw. Q1-Q4 2022 (26,28–30)					57,54		56,79		56,98

Tabelle S9: Beispielhafte Berechnung des Honorars eines HNO-Arztes für allergologische Diagnostik und SLIT in 2022 in der KVB. Der DF wurde zur Berechnung der abgesenkten RLV-Vergütung verwendet. Zum Vergleich der Honorare ist auch das DHPB aufgeführt.

Honorar-Übersicht von Diagnostik und dreijähriger AIT in der KVB

**Anzahl Quartale**	**Quartale**	**DHPB (€)**	**Vergütung in einer budgeteinhaltenden Praxis (€)**		**Vergütung in einer budgetüberschreitenden Praxis (€)**	
			**Diagnostik (nur in Q1) und SCIT**	**Diagnostik (nur in Q1) und SLIT**	**Diagnostik (nur in Q1) und SCIT**	**Diagnostik (nur in Q1) und SLIT**
1	Q1	57,54	138,98	90,10	92,10	64,87
3	Q2-4	56,79	65,45	26,93	51,46	26,93
8	Q5-12	56,98	72,77	34,25	58,78	34,25
**12**	**Q1-12**	**683,75**	**917,50**	**444,85**	**716,67**	**419,62**

Tabelle S10: Darstellung des maximal erhältlichen und des abgesenkten Honorars (gesamt und pro Quartal) eines HNO-Arztes für allergologische Diagnostik und eine dreijährige AIT in der KVB bei einem adhärenten Patienten. Zum Vergleich der Honorare ist auch das Quartals- und Gesamt-DHPB dargestellt.

Berlin

Vergütung der AIT in einer budgetüberschreitenden HNO-Praxis

Pos.	EBM Ziffer	Leistung (SCIT)	Leistung (€)	N	Gesamt (€)	N	Gesamt (€)	N	Gesamt (€)
				Q1		Q2-4		Q5-12	
1	09211	Grundpauschale 6.- 59. Lebensjahr	23,10	1	23,10	1	23,10	1	23,10
2	09220	Zuschlag für die HNO Grundversorgung	3,04	0	0,00	1	3,04	1	3,04
3	09222	Zuschlag zu der GOP 09220	0,79	0	0,00	1	0,79	1	0,79
4	30100	Anamnese/Beratung	7,32	4	29,28	0	0,00	1	7,32
5	30111	Diagnostik	24,79	1	24,79	0	0,00	0	0,00
6	40351	Kostenpauschale Sachkosten	5,50	1	5,50	0	0,00	0	0,00
7	30120	Rhinomanometr. Provokationstest	7,44	1	7,44	0	0,00	0	0,00
8	30130	Injektion Grund- und Fortsetzungs-behandlung	11,49	3	34,47	3	34,47	3	34,47
9	30131	Zuschlag für die 2. Injektion	9,01	1	9,01	0	0,00	0	0,00
**EBM-Vergütung BEV (Summe Pos 1,2,4,5,7,8,9)**					**128,08**		**60,61**		**67,93**
**Pauschalen und extrabudgetäre Leistungen (Summe Pos. 3,6 und evtl. FL)**					**17,08**		**9,97**		**9,97**
Durchschnittlicher RLV-Fallwert					32,18		32,18		32,18
					**Gesamt (€)**		**Gesamt (€)**		**Gesamt (€)**
AV RLV - Überschreitung zum durchschnittlichen Fallwert (DF) mit 15% berechnet					46,57		36,44		37,54
Pauschalen und extrabudgetäre Leistungen (Summe Pos. 3,6 und evtl. FL)					17,08		9,97		9,97
**Gesamthonorar**					**63,65**		**46,41**		**47,51**
DHPB in Q1, Q2-Q4 bzw. Q1-Q4 2022 (26,28–30)					52,30		51,75		51,89

Tabelle S11: Beispielhafte Berechnung des Honorars eines HNO-Arztes für allergologische Diagnostik und SCIT in 2022 in der KVBerlin. Der DF wurde zur Berechnung der abgesenkten RLV-Vergütung verwendet. Zum Vergleich der Honorare ist auch das DHPB aufgeführt.

Pos.	EBM Ziffer	Leistung (SLIT)	Leistung (€)	N	Gesamt (€)	N	Gesamt (€)	N	Gesamt (€)
				Q1		Q2-4		Q5-12	
1	09211	Grundpauschale 6.-59. Lebensjahr	23,10	1	23,10	1	23,10	1	23,10
2	09220	Zuschlag für die HNO Grundversorgung	3,04	0	0,00	1	3,04	1	3,04
3	09222	Zuschlag zu der GOP 09220	0,79	0	0,00	1	0,79	1	0,79
4	30100	Anamnese/Beratung	7,32	4	29,28	0	0,00	1	7,32
5	30111	Diagnostik	24,79	1	24,79	0	0,00	0	0,00
6	40351	Kostenpauschale Sachkosten	5,50	1	5,50	0	0,00	0	0,00
7	30120	Rhinomanometr. Provokationstest	7,44	1	7,44	0	0,00	0	0,00
**EBM-Vergütung RLV (Summe Pos 1,2,4,5,7)**					**84,60**		**26,14**		**33,46**
**Pauschalen und extrabudgetäre Leistungen (Summe Pos. 3,6 und evtl. FL)**					**15,83**		**11,12**		**11,12**
Durchschnittlicher RLV-Fallwert					32,18		32,18		32,18
					**Gesamt (€)**		**Gesamt (€)**		**Gesamt (€)**
Abgesenkte Vergütung (AV) RLV - Überschreitung zum durchschnittlichen Fallwert (DF) mit 15% berechnet					40,04		26,14		32,37
Pauschalen und extrabudgetäre Leistungen (Summe Pos. 3,6 und evtl. FL)					15,83		11,12		11,12
**Gesamthonorar**					**55,87**		**37,26**		**43,49**
DHPB in Q1, Q2-Q4 bzw. Q1-Q4 2022 (26,28–30)					52,30		51,75		51,89

Tabelle S12: Beispielhafte Berechnung des Honorars eines HNO-Arztes für allergologische Diagnostik und SLIT in 2022 in der KVBerlin. Der DF wurde zur Berechnung der abgesenkten RLV-Vergütung verwendet. Zum Vergleich der Honorare ist auch das DHPB aufgeführt.

Honorar-Übersicht von Diagnostik und dreijähriger AIT in der KVBerlin

Anzahl Quartale	Quartale	DHPB (€)	Vergütung in einer budgeteinhaltenden Praxis (€)		Vergütung in einer budgetüberschreitenden Praxis (€)	
			Diagnostik (nur in Q1) und SCIT	Diagnostik (nur in Q1) und SLIT	Diagnostik (nur in Q1) und SCIT	Diagnostik (nur in Q1) und SLIT
1	Q1	52,3	145,16	100,43	63,65	55,87
3	Q2-4	51,75	70,58	37,26	46,41	37,26
8	Q5-12	51,89	77,90	44,58	47,51	43,49
GOP 91130			30,00	30,00	30,00	30,00
**12**	**Q1-12**	**622,67**	**1010,14**	**598,83**	**612,98**	**545,57**

Tabelle S13: Darstellung des maximal erhältlichen und des abgesenkten Honorars (gesamt und pro Quartal) eines HNO-Arztes für allergologische Diagnostik und eine dreijährige AIT in der KVBerlin bei einem adhärenten Patienten. Zum Vergleich der Honorare ist auch das Quartals- und Gesamt-DHPB dargestellt.

Brandenburg

Vergütung der AIT in einer budgetüberschreitenden HNO-Praxis

**Pos.**	**EBM Ziffer**	**Leistung (SCIT)**	**Leistung (€)**	**N**	**Gesamt (€)**	**N**	**Gesamt (€)**	**N**	**Gesamt (€)**
				**Q1**		**Q2-4**		**Q5-12**	
1	09211	Grundpauschale 6.- 59. Lebensjahr	23,10	1	23,10	1	23,10	1	23,10
2	09220	Zuschlag für die HNO Grundversorgung	3,04	0	0,00	1	3,04	1	3,04
3	09222	Zuschlag zu der GOP 09220	0,79	0	0,00	1	0,79	1	0,79
4	30100	Anamnese/Beratung	7,32	4	29,28	0	0,00	1	7,32
5	30111	Diagnostik	24,79	1	24,79	0	0,00	0	0,00
6	40351	Kostenpauschale Sachkosten	5,50	1	5,50	0	0,00	0	0,00
7	30120	Rhinomanometr. Provokationstest	7,44	1	7,44	0	0,00	0	0,00
8	30130	Injektion Grund- und Fortsetzungs-behandlung	11,49	3	34,47	3	34,47	3	34,47
9	30131	Zuschlag für die 2. Injektion	9,01	1	9,01	0	0,00	0	0,00
**EBM-Vergütung RLV (Summe Pos 1,2,4,5,7,8,9)**					**128,08**		**60,61**		**67,93**
**Pauschalen und extrabudgetäre Leistungen (Summe Pos. 3,6 und evtl. FL)**					**25,50**		**15,79**		**15,79**
Durchschnittlicher RLV-Fallwert					27,79		27,79		27,79
					**Gesamt (€)**		**Gesamt (€)**		**Gesamt (€)**
AV RLV - Überschreitung zum durchschnittlichen Fallwert (DF) mit 15% berechnet					42,83		32,71		33,81
Pauschalen und extrabudgetäre Leistungen (Summe Pos. 3,6 und evtl. FL)					25,50		15,79		15,79
**Gesamthonorar**					**68,33**		**48,50**		**49,60**
DHPB in Q1, Q2-Q4 bzw. Q1-Q4 2022 (26,28–30)					48,55		48,45		48,47

Tabelle S14: Beispielhafte Berechnung des Honorars eines HNO-Arztes für allergologische Diagnostik und SCIT in 2022 in der KVBB. Der DF wurde zur Berechnung der abgesenkten RLV-Vergütung verwendet. Zum Vergleich der Honorare ist auch das DHPB aufgeführt.

Pos.	EBM Ziffer	Leistung (SLIT)	Leistung (€)	N	Gesamt (€)	N	Gesamt (€)	N	Gesamt (€)
				Q1		Q2-4		Q5-12	
1	09211	Grundpauschale 6.- 59. Lebensjahr	23,10	1	23,10	1	23,10	1	23,10
2	09220	Zuschlag für die HNO Grundversorgung	3,04	0	0,00	1	3,04	1	3,04
3	09222	Zuschlag zu der GOP 09220	0,79	0	0,00	1	0,79	1	0,79
4	30100	Anamnese/Beratung	7,32	4	29,28	0	0,00	1	7,32
5	30111	Diagnostik	24,79	1	24,79	0	0,00	0	0,00
6	40351	Kostenpauschale Sachkosten	5,50	1	5,50	0	0,00	0	0,00
7	30120	Rhinomanometr. Provokationstest	7,44	1	7,44	0	0,00	0	0,00
**EBM-Vergütung RLV (Summe Pos 1,2,4,5,7)**					**84,60**		**26,14**		**33,46**
**Pauschalen und extrabudgetäre Leistungen (Summe Pos. 3,6 und evtl. FL)**					**5,50**		**0,79**		**0,79**
Durchschnittlicher RLV-Fallwert					27,79		27,79		27,79
					**Gesamt (€)**		**Gesamt (€)**		**Gesamt (€)**
AV RLV - Überschreitung zum durchschnittlichen Fallwert (DF) mit 15% berechnet					36,31		26,14		28,64
Pauschalen und extrabudgetäre Leistungen (Summe Pos. 3,6 und evtl. FL)					5,50		0,79		0,79
**Gesamthonorar**					**41,81**		**26,93**		**29,43**
DHPB in Q1, Q2-Q4 bzw. Q1-Q4 2022 (26,28–30)					48,55		48,45		48,47

Tabelle S15: Beispielhafte Berechnung des Honorars eines HNO-Arztes für allergologische Diagnostik und SLIT in 2022 in der KVBB. Der DF wurde zur Berechnung der abgesenkten RLV-Vergütung verwendet. Zum Vergleich der Honorare ist auch das DHPB aufgeführt.

Honorar-Übersicht von Diagnostik und dreijähriger AIT in der KVBB

Anzahl Quartale	Quartale	DHPB (€)	Vergütung in einer budgeteinhaltenden Praxis (€)		Vergütung in einer budgetüberschreitenden Praxis (€)	
			Diagnostik (nur in Q1) und SCIT	Diagnostik (nur in Q1) und SLIT	Diagnostik (nur in Q1) und SCIT	Diagnostik (nur in Q1) und SLIT
1	Q1	48,55	153,58	90,10	68,33	41,81
3	Q2-4	48,45	76,40	26,93	48,50	26,93
8	Q5-12	48,47	83,72	34,25	49,60	29,43
**12**	**Q1-12**	**581,66**	**1052,58**	**444,87**	**610,64**	**358,02**

Tabelle S16: Darstellung des maximal erhältlichen und des abgesenkten Honorars (gesamt und pro Quartal) eines HNO-Arztes für allergologische Diagnostik und eine dreijährige AIT in der KVBB bei einem adhärenten Patienten. Zum Vergleich der Honorare ist auch das Quartals- und Gesamt-DHPB dargestellt.

Bremen

Vergütung der AIT in einer budgetüberschreitenden HNO-Praxis

Pos.	EBM Ziffer	Leistung (SCIT)	Leistung (€)	N	Gesamt (€)	N	Gesamt (€)	N	Gesamt (€)
				Q1		Q2-4		Q5-12	
1	09211	Grundpauschale 6.- 59. Lebensjahr	23,10	1	23,10	1	23,10	1	23,10
2	09220	Zuschlag für die HNO Grundversorgung	3,04	0	0,00	1	3,04	1	3,04
3	09222	Zuschlag zu der GOP 09220	0,79	0	0,00	1	0,79	1	0,79
4	30100	Anamnese/Beratung	7,32	4	29,28	0	0,00	1	7,32
5	30111	Diagnostik	24,79	1	24,79	0	0,00	0	0,00
6	40351	Kostenpauschale Sachkosten	5,50	1	5,50	0	0,00	0	0,00
7	30120	Rhinomanometr. Provokationstest	7,44	1	7,44	0	0,00	0	0,00
8	30130	Injektion Grund- und Fortsetzungs-behandlung	11,49	3	34,47	3	34,47	3	34,47
9	30131	Zuschlag für die 2. Injektion	9,01	1	9,01	0	0,00	0	0,00
**EBM-Vergütung RLV (Summe Pos 1,2,4,5,7,8,9)**					**128,08**		**60,61**		**67,93**
**Pauschalen und extrabudgetäre Leistungen (Summe Pos. 3,6 und evtl. FL)**					**15,52**		**10,81**		**10,81**
Durchschnittlicher RLV-Fallwert					28,41		28,41		28,41
					**Gesamt (€)**		**Gesamt (€)**		**Gesamt (€)**
AV RLV - Überschreitung zum durchschnittlichen Fallwert (DF) mit 15% berechnet					43,36		33,24		34,34
Pauschalen und extrabudgetäre Leistungen (Summe Pos. 3,6 und evtl. FL)					15,52		10,81		10,81
**Gesamthonorar**					**58,88**		**44,05**		**45,15**
DHPB in Q1, Q2-Q4 bzw. Q1-Q4 2022 (26,28–30)					49,64		48,43		48,74

Tabelle S17: Beispielhafte Berechnung des Honorars eines HNO-Arztes für allergologische Diagnostik und SCIT in 2022 in der KVHB. Der DF wurde zur Berechnung der abgesenkten RLV-Vergütung verwendet. Zum Vergleich der Honorare ist auch das DHPB aufgeführt.

Pos.	EBM Ziffer	Leistung (SLIT)	Leistung (€)	N	Gesamt (€)	N	Gesamt (€)	N	Gesamt (€)
				Q1		Q2-4		Q5-12	
1	09211	Grundpauschale 6.- 59. Lebensjahr	23,10	1	23,10	1	23,10	1	23,10
2	09220	Zuschlag für die HNO Grundversorgung	3,04	0	0,00	1	3,04	1	3,04
3	09222	Zuschlag zu der GOP 09220	0,79	0	0,00	1	0,79	1	0,79
4	30100	Anamnese/Beratung	7,32	4	29,28	0	0,00	1	7,32
5	30111	Diagnostik	24,79	1	24,79	0	0,00	0	0,00
6	40351	Kostenpauschale Sachkosten	5,50	1	5,50	0	0,00	0	0,00
7	30120	Rhinomanometr. Provokationstest	7,44	1	7,44	0	0,00	0	0,00
**EBM-Vergütung RLV (Summe Pos 1,2,4,5,7)**					**84,60**		**26,14**		**33,46**
**Pauschalen und extrabudgetäre Leistungen (Summe Pos. 3,6 und evtl. FL)**					**5,50**		**0,79**		**0,79**
Durchschnittlicher RLV-Fallwert					28,41		28,41		28,41
					**Gesamt (€)**		**Gesamt (€)**		**Gesamt (€)**
AV RLV - Überschreitung zum durchschnittlichen Fallwert (DF) mit 15% berechnet					36,84		26,14		29,17
Pauschalen und extrabudgetäre Leistungen (Summe Pos. 3,6 und evtl. FL)					5,50		0,79		0,79
**Gesamthonorar**					**42,34**		**26,93**		**29,96**
DHPB in Q1, Q2-Q4 bzw. Q1-Q4 2022 (26,28–30)					49,64		48,43		48,74

Tabelle S18: Beispielhafte Berechnung des Honorars eines HNO-Arztes für allergologische Diagnostik und SLIT in 2022 in der KVHB. Der DF wurde zur Berechnung der abgesenkten RLV-Vergütung verwendet. Zum Vergleich der Honorare ist auch das DHPB aufgeführt.

Honorar-Übersicht von Diagnostik und dreijähriger AIT in der KVHB

Anzahl Quartale	Quartale	DHPB (€)	Vergütung in einer budgeteinhaltenden Praxis (€)		Vergütung in einer budgetüberschreitenden Praxis (€)	
			**Diagnostik (nur in Q1) und SCIT**	**Diagnostik (nur in Q1) und SLIT**	**Diagnostik (nur in Q1) und SCIT**	**Diagnostik (nur in Q1) und SLIT**
1	Q1	49,64	143,60	90,10	58,88	42,34
3	Q2-4	48,43	71,42	26,93	44,05	26,93
8	Q5-12	48,74	78,74	34,25	45,15	29,96
GOP 99055			70,00		70,00	
**12**	**Q1-12**	**584,85**	**1057,82**	**444,87**	**622,21**	**362,77**

Tabelle S19: Darstellung des maximal erhältlichen und des abgesenkten Honorars (gesamt und pro Quartal) eines HNO-Arztes für allergologische Diagnostik und eine dreijährige AIT in der KVHB bei einem adhärenten Patienten. Zum Vergleich der Honorare ist auch das Quartals- und Gesamt-DHPB dargestellt.

Hamburg

Vergütung der AIT in einer budgetüberschreitenden HNO-Praxis

Pos.	EBM Ziffer	Leistung (SCIT)	Leistung (€)	N	Gesamt (€)	N	Gesamt (€)	N	Gesamt (€)
				Q1		Q2-4		Q5-12	
1	09211	Grundpauschale 6.- 59. Lebensjahr	23,10	1	23,10	1	23,10	1	23,10
2	09220	Zuschlag für die HNO Grundversorgung	3,04	0	0,00	1	3,04	1	3,04
3	09222	Zuschlag zu der GOP 09220	0,79	0	0,00	1	0,79	1	0,79
4	30100	Anamnese/Beratung	7,32	4	29,28	0	0,00	1	7,32
5	30111	Diagnostik	24,79	1	24,79	0	0,00	0	0,00
6	40351	Kostenpauschale Sachkosten	5,50	1	5,50	0	0,00	0	0,00
7	30120	Rhinomanometr. Provokationstest	7,44	1	7,44	0	0,00	0	0,00
8	30130	Injektion Grund- und Fortsetzungs-behandlung	11,49	3	34,47	3	34,47	3	34,47
9	30131	Zuschlag für die 2. Injektion	9,01	1	9,01	0	0,00	0	0,00
**EBM-Vergütung RLV (Summe Pos 1,2,4,5,7,8,9)**					**130,18**		**61,60**		**69,04**
**Pauschalen und extrabudgetäre Leistungen (Summe Pos. 3,6 und evtl. FL)**					**5,50**		**0,79**		**0,79**
Durchschnittliche Garantiequote					82%		82%		82%
					**Gesamt (€)**		**Gesamt (€)**		**Gesamt (€)**
AV RLV - Überschreitung zum durchschnittlichen Fallwert (DF) mit 15% berechnet					106,75		50,51		56,61
Pauschalen und extrabudgetäre Leistungen (Summe Pos. 3,6 und evtl. FL)					5,50		0,79		0,79
**Gesamthonorar**					**112,25**		**51,30**		**57,40**
DHPB in Q1, Q2-Q4 bzw. Q1-Q4 2022 (26,28–30)					58,63		57,87		58,06

Tabelle S20: Beispielhafte Berechnung des Honorars eines HNO-Arztes für allergologische Diagnostik und SCIT in 2022 in der KVHH. Der DF wurde zur Berechnung der abgesenkten RLV-Vergütung verwendet. Zum Vergleich der Honorare ist auch das DHPB aufgeführt. Der Punktwert zur Berechnung der regionalen EUR-Gebührenordnung beträgt 11,4494 Cent. Er ergibt sich als regionaler Punktwert aus dem um einen Zuschlag von 1,626 Prozent (0,1832 Cent) erhöhten bundeseinheitlichen Punktwert von 11,2662 Cent als Orientierungswert (15).

Pos.	EBM Ziffer	Leistung (SLIT)	Leistung (€)	N	Gesamt (€)	N	Gesamt (€)	N	Gesamt (€)
				Q1		Q2-4		Q5-12	
1	09211	Grundpauschale 6.- 59. Lebensjahr	23,10	1	23,10	1	23,10	1	23,10
2	09220	Zuschlag für die HNO Grundversorgung	3,04	0	0,00	1	3,04	1	3,04
3	09222	Zuschlag zu der GOP 09220	0,79	0	0,00	1	0,79	1	0,79
4	30100	Anamnese/Beratung	7,32	4	29,28	0	0,00	1	7,32
5	30111	Diagnostik	24,79	1	24,79	0	0,00	0	0,00
6	40351	Kostenpauschale Sachkosten	5,50	1	5,50	0	0,00	0	0,00
7	30120	Rhinomanometr. Provokationstest	7,44	1	7,44	0	0,00	0	0,00
**EBM-Vergütung RLV (Summe Pos 1,2,4,5,7)**					**85,97**		**26,56**		**34,00**
**Pauschalen und extrabudgetäre Leistungen (Summe Pos. 3,6 und evtl. FL)**					**5,50**		**0,79**		**0,79**
Durchschnittliche Garantiequote					82%		82%		82%
					**Gesamt (€)**		**Gesamt (€)**		**Gesamt (€)**
AV RLV - Überschreitung zum durchschnittlichen Fallwert (DF) mit 15% berechnet					70,50		21,78		27,88
Pauschalen und extrabudgetäre Leistungen (Summe Pos. 3,6 und evtl. FL)					5,50		0,79		0,79
**Gesamthonorar**					**76,00**		**22,57**		**28,67**
DHPB in Q1, Q2-Q4 bzw. Q1-Q4 2022 (26,28–30)					58,63		57,87		58,06

Tabelle S21: Beispielhafte Berechnung des Honorars eines HNO-Arztes für allergologische Diagnostik und SLIT in 2022 in der KVHH. Der DF wurde zur Berechnung der abgesenkten RLV-Vergütung verwendet. Zum Vergleich der Honorare ist auch das DHPB aufgeführt. Der Punktwert zur Berechnung der regionalen EUR-Gebührenordnung beträgt 11,4494 Cent. Er ergibt sich als regionaler Punktwert aus dem um einen Zuschlag von 1,626 Prozent (0,1832 Cent) erhöhten bundeseinheitlichen Punktwert von 11,2662 Cent als Orientierungswert (15).

Honorar-Übersicht von Diagnostik und dreijähriger AIT in der KVHH

Anzahl Quartale	Quartale	DHPB (€)	Vergütung in einer budgeteinhaltenden Praxis (€)		Vergütung in einer budgetüberschreitenden Praxis (€)	
			Diagnostik (nur in Q1) und SCIT	Diagnostik (nur in Q1) und SLIT	Diagnostik (nur in Q1) und SCIT	Diagnostik (nur in Q1) und SLIT
1	Q1	58,63	135,68	91,47	112,25	76,00
3	Q2-4	57,87	62,39	27,35	51,30	22,57
8	Q5-12	58,06	69,83	34,79	57,40	28,67
GOP 98000			100,00		100,00	
**12**	**Q1-12**	**696,72**	**981,46**	**451,87**	**825,35**	**373,09**

Tabelle S22: Darstellung des maximal erhältlichen und des abgesenkten Honorars (gesamt und pro Quartal) eines HNO-Arztes für allergologische Diagnostik und eine dreijährige AIT in der KVHH bei einem adhärenten Patienten. Zum Vergleich der Honorare ist auch das Quartals- und Gesamt-DHPB dargestellt.

Hessen

Vergütung der AIT in einer budgetüberschreitenden HNO-Praxis

Pos.	EBM Ziffer	Leistung (SCIT)	Leistung (€)	N	Gesamt (€)	N	Gesamt (€)	N	Gesamt (€)
					**Q1**		**Q2-4**		**Q5-12**
1	09211	Grundpauschale 6.- 59. Lebensjahr	23,10	1	23,10	1	23,10	1	23,10
2	09220	Zuschlag für die HNO Grundversorgung	3,04	0	0,00	1	3,04	1	3,04
3	09222	Zuschlag zu der GOP 09220	0,79	0	0,00	1	0,79	1	0,79
4	30100	Anamnese/Beratung	7,32	4	29,28	0	0,00	1	7,32
5	30111	Diagnostik	24,79	1	24,79	0	0,00	0	0,00
6	40351	Kostenpauschale Sachkosten	5,50	1	5,50	0	0,00	0	0,00
7	30120	Rhinomanometr. Provokationstest	7,44	1	7,44	0	0,00	0	0,00
8	30130	Injektion Grund- und Fortsetzungs-behandlung	11,49	3	34,47	3	34,47	3	34,47
9	30131	Zuschlag für die 2. Injektion	9,01	1	9,01	0	0,00	0	0,00
**EBM-Vergütung RLV (Summe Pos 1,2,4,5,7,8,9)**					**128,08**		**60,61**		**67,93**
**Pauschalen und extrabudgetäre Leistungen (Summe Pos. 3,6 und evtl. FL)**					**15,96**		**4,15**		**4,15**
Durchschnittlicher RLV-Fallwert					50,19		50,19		50,19
					**Gesamt (€)**		**Gesamt (€)**		**Gesamt (€)**
AV RLV - Überschreitung zum durchschnittlichen Fallwert (DF) mit 15% berechnet					67,37		51,75		52,85
Pauschalen und extrabudgetäre Leistungen (Summe Pos. 3,6 und evtl. FL)					15,96		4,15		4,15
**Gesamthonorar**					**83,33**		**55,90**		**57,00**
DHPB in Q1, Q2-Q4 bzw. Q1-Q4 2022 (26,28–30)					51,86		52,50		52,34

Tabelle S23: Beispielhafte Berechnung des Honorars eines HNO-Arztes für allergologische Diagnostik und SCIT in 2022 in der KVH. Der DF wurde zur Berechnung der abgesenkten RLV-Vergütung verwendet. Zum Vergleich der Honorare ist auch das DHPB aufgeführt.

Pos.	EBM Ziffer	Leistung (SLIT)	Leistung (€)	N	Gesamt (€)	N	Gesamt (€)	N	Gesamt (€)
					Q1		Q2-4		Q5-12
1	09211	Grundpauschale 6.- 59. Lebensjahr	23,10	1	23,10	1	23,10	1	23,10
2	09220	Zuschlag für die HNO Grundversorgung	3,04	0	0,00	1	3,04	1	3,04
3	09222	Zuschlag zu der GOP 09220	0,79	0	0,00	1	0,79	1	0,79
4	30100	Anamnese/Beratung	7,32	4	29,28	0	0,00	1	7,32
5	30111	Diagnostik	24,79	1	24,79	0	0,00	0	0,00
6	40351	Kostenpauschale Sachkosten	5,50	1	5,50	0	0,00	0	0,00
7	30120	Rhinomanometr. Provokationstest	7,44	1	7,44	0	0,00	0	0,00
**EBM-Vergütung RLV (Summe Pos 1,2,4,5,7)**					**84,60**		**26,14**		**33,46**
**Pauschalen und extrabudgetäre Leistungen (Summe Pos. 3,6 und evtl. FL)**					**11,75**		**0,79**		**0,79**
Durchschnittlicher RLV-Fallwert					50,19		50,19		50,19
					**Gesamt (€)**		**Gesamt (€)**		**Gesamt (€)**
AV RLV - Überschreitung zum durchschnittlichen Fallwert (DF) mit 15% berechnet					60,85		26,14		33,46
Pauschalen und extrabudgetäre Leistungen (Summe Pos. 3,6 und evtl. FL)					11,75		0,79		0,79
**Gesamthonorar**					**72,60**		**26,93**		**34,25**
DHPB in Q1, Q2-Q4 bzw. Q1-Q4 2022 (26,28–30)					51,86		52,50		52,34

Tabelle S24: Beispielhafte Berechnung des Honorars eines HNO-Arztes für allergologische Diagnostik und SLIT in 2022 in der KVH. Der DF wurde zur Berechnung der abgesenkten RLV-Vergütung verwendet. Zum Vergleich der Honorare ist auch das DHPB aufgeführt.

Honorar-Übersicht von Diagnostik und dreijähriger AIT in der KVH

Anzahl Quartale	Quartale	DHPB (€)	Vergütung in einer budgeteinhaltenden Praxis (€)		Vergütung in einer budgetüberschreitenden Praxis (€)	
			Diagnostik (nur in Q1) und SCIT	Diagnostik (nur in Q1) und SLIT	Diagnostik (nur in Q1) und SCIT	Diagnostik (nur in Q1) und SLIT
1	Q1	51,86	144,04	96,35	83,33	72,60
3	Q2-4	52,50	64,76	26,93	55,90	26,93
8	Q5-12	52,34	72,08	34,25	57,00	34,25
**12**	**Q1-12**	**628,08**	**915,00**	**451,12**	**707,04**	**427,37**

Tabelle S25: Darstellung des maximal erhältlichen und des abgesenkten Honorars (gesamt und pro Quartal) eines HNO-Arztes für allergologische Diagnostik und eine dreijährige AIT in der KVH bei einem adhärenten Patienten. Zum Vergleich der Honorare ist auch das Quartals- und Gesamt-DHPB dargestellt.

Niedersachsen

Vergütung der AIT in einer budgetüberschreitenden HNO-Praxis

Pos.	EBM Ziffer	Leistung (SCIT)	Leistung (€)	N	Gesamt (€)	N	Gesamt (€)	N	Gesamt (€)
				Q1		Q2-4		Q5-12	
1	09211	Grundpauschale 6.- 59. Lebensjahr	23,10	1	23,10	1	23,10	1	23,10
2	09220	Zuschlag für die HNO Grundversorgung	3,04	0	0,00	1	3,04	1	3,04
3	09222	Zuschlag zu der GOP 09220	0,79	0	0,00	1	0,79	1	0,79
4	30100	Anamnese/Beratung	7,32	4	29,28	0	0,00	1	7,32
5	30111	Diagnostik	24,79	1	24,79	0	0,00	0	0,00
6	40351	Kostenpauschale Sachkosten	5,50	1	5,50	0	0,00	0	0,00
7	30120	Rhinomanometr. Provokationstest	7,44	1	7,44	0	0,00	0	0,00
8	30130	Injektion Grund- und Fortsetzungs-behandlung	11,49	3	34,47	3	34,47	3	34,47
9	30131	Zuschlag für die 2. Injektion	9,01	1	9,01	0	0,00	0	0,00
**EBM-Vergütung RLV (Summe Pos. 1,2)**					**23,10**		**26,14**		**26,14**
**EBM-Vergütung QZV Diagnostik (Summe Pos. 4,5,7)**					**61,50**		**0,00**		**7,32**
**EBM-Vergütung QZV Hyposensibilisierung (Summe Pos. 8,9)**					**43,49**		**34,47**		**34,47**
**Pauschalen und extrabudgetäre Leistungen (Summe Pos. 3,6 und evtl. FL)**					**5,50**		**0,79**		**0,79**
Durchschnittlicher RLV-Fallwert					25,32		25,32		25,32
Durchschnittlicher QZV Diagnostik-Fallwert					8,63		8,63		8,63
Durchschnittlicher QZV Hyposens.-Fallwert					10,49		10,49		10,49
					**Gesamt (€)**		**Gesamt (€)**		**Gesamt (€)**
AV RLV - Überschreitung zum DF mit 15% berechnet					23,10		25,44		25,44
AV QZV Diagnostik - Überschreitung zum DF mit 15% berechnet					16,56		0,00		7,32
AV QZV Hyposens. - Überschreitung zum DF mit 15% berechnet					15,44		14,09		14,09
Pauschalen und extrabudgetäre Leistungen (Summe Pos. 3,6 und evtl. FL)					5,50		0,79		0,79
**Gesamthonorar**					**60,60**		**40,32**		**47,64**
DHPB in Q1, Q2-Q4 bzw. Q1-Q4 2022 (26,28–30)					50,60		50,27		50,35

Tabelle S26: Beispielhafte Berechnung des Honorars eines HNO-Arztes für allergologische Diagnostik und SCIT in 2022 in der KVN. Der DF wurde zur Berechnung der abgesenkten RLV-Vergütung verwendet. Zum Vergleich der Honorare ist auch das DHPB aufgeführt.

Pos.	EBM Ziffer	Leistung (SLIT)	Leistung (€)	N	Gesamt (€)	N	Gesamt (€)	N	Gesamt (€)
				Q1		Q2-4		Q5-12	
1	09211	Grundpauschale 6.- 59. Lebensjahr	23,10	1	23,10	1	23,10	1	23,10
2	09220	Zuschlag für die HNO Grundversorgung	3,04	0	0,00	1	3,04	1	3,04
3	09222	Zuschlag zu der GOP 09220	0,79	0	0,00	1	0,79	1	0,79
4	30100	Anamnese/Beratung	7,32	4	29,28	0	0,00	1	7,32
5	30111	Diagnostik	24,79	1	24,79	0	0,00	0	0,00
6	40351	Kostenpauschale Sachkosten	5,50	1	5,50	0	0,00	0	0,00
7	30120	Rhinomanometr. Provokationstest	7,44	1	7,44	0	0,00	0	0,00
**EBM-Vergütung RLV (Summe Pos. 1,2)**					**23,10**		**26,14**		**26,14**
**EBM-Vergütung QZV Diagnostik (Summe Pos. 4,5,7)**					**61,50**		**0,00**		**7,32**
**Pauschalen und extrabudgetäre Leistungen (Summe Pos. 3,6 und evtl. FL)**					**5,50**		**0,79**		**0,79**
Durchschnittlicher RLV-Fallwert					25,32		25,32		25,32
Durchschnittlicher QZV Diagnostik-Fallwert					8,63		8,63		8,63
					Gesamt (€)		Gesamt (€)		Gesamt (€)
AV RLV - Überschreitung zum DF mit 15% berechnet					23,10		25,44		25,44
AV QZV Diagnostik - Überschreitung zum DF mit 15% berechnet					16,56		0,00		7,32
Pauschalen und extrabudgetäre Leistungen (Summe Pos. 3,6 und evtl. FL)					5,50		0,79		0,79
**Gesamthonorar**					**45,16**		**26,23**		**33,55**
DHPB in Q1, Q2-Q4 bzw. Q1-Q4 2022 (26,28–30)					50,60		50,27		50,35

Tabelle S27: Beispielhafte Berechnung des Honorars eines HNO-Arztes für allergologische Diagnostik und SLIT in 2022 in der KVN. Der DF wurde zur Berechnung der abgesenkten RLV-Vergütung verwendet. Zum Vergleich der Honorare ist auch das DHPB aufgeführt.

Honorar-Übersicht von Diagnostik und dreijähriger AIT in der KVN

Anzahl Quartale	Quartale	DHPB (€)	Vergütung in einer budgeteinhaltenden Praxis (€)		Vergütung in einer budgetüberschreitenden Praxis (€)	
			Diagnostik (nur in Q1) und SCIT	Diagnostik (nur in Q1) und SLIT	Diagnostik (nur in Q1) und SCIT	Diagnostik (nur in Q1) und SLIT
1	Q1	50,60	133,58	90,10	60,60	45,16
3	Q2-4	50,27	61,40	26,93	40,32	26,23
8	Q5-12	50,35	68,72	34,25	47,64	33,55
**12**	**Q1-12**	**604,21**	**867,58**	**444,87**	**562,69**	**392,28**

Tabelle S28: Darstellung des maximal erhältlichen und des abgesenkten Honorars (gesamt und pro Quartal) eines HNO-Arztes für allergologische Diagnostik und eine dreijährige AIT in der KVN bei einem adhärenten Patienten. Zum Vergleich der Honorare ist auch das Quartals- und Gesamt-DHPB dargestellt.

Nordrhein

Vergütung der AIT in einer budgetüberschreitenden HNO-Praxis

Pos.	EBM Ziffer	Leistung (SCIT)	Leistung (€)	N	Gesamt (€)	N	Gesamt (€)	N	Gesamt (€)
				Q1		Q2-4		Q5-12	
1	09211	Grundpauschale 6.- 59. Lebensjahr	23,10	1	23,10	1	23,10	1	23,10
2	09220	Zuschlag für die HNO Grundversorgung	3,04	0	0,00	1	3,04	1	3,04
3	09222	Zuschlag zu der GOP 09220	0,79	0	0,00	1	0,79	1	0,79
4	30100	Anamnese/Beratung	7,32	4	29,28	0	0,00	1	7,32
5	30111	Diagnostik	24,79	1	24,79	0	0,00	0	0,00
6	40351	Kostenpauschale Sachkosten	5,50	1	5,50	0	0,00	0	0,00
7	30120	Rhinomanometr. Provokationstest	7,44	1	7,44	0	0,00	0	0,00
8	30130	Injektion Grund- und Fortsetzungs-behandlung	11,49	3	34,47	3	34,47	3	34,47
9	30131	Zuschlag für die 2. Injektion	9,01	1	9,01	0	0,00	0	0,00
**EBM-Vergütung RLV (Summe Pos 1,2,4,5,7,8,9)**					**128,08**		**60,61**		**67,93**
**Pauschalen und extrabudgetäre Leistungen (Summe Pos. 3,6 und evtl. FL)**					**55,10**		**13,24**		**13,24**
Durchschnittlicher RLV-Fallwert					25,63		25,63		25,63
					**Gesamt (€)**		**Gesamt (€)**		**Gesamt (€)**
AV RLV - Überschreitung zum durchschnittlichen Fallwert (DF) mit 15% berechnet					41,00		30,88		31,98
Pauschalen und extrabudgetäre Leistungen (Summe Pos. 3,6 und evtl. FL)					55,10		13,24		13,24
**Gesamthonorar**					**96,10**		**44,11**		**45,21**
DHPB in Q1, Q2-Q4 bzw. Q1-Q4 2022 (26,28–30)					46,99		46,20		46,40

Tabelle S29: Beispielhafte Berechnung des Honorars eines HNO-Arztes für allergologische Diagnostik und SCIT in 2022 in der KVNo. Der DF wurde zur Berechnung der abgesenkten RLV-Vergütung verwendet. Zum Vergleich der Honorare ist auch das DHPB aufgeführt.

Pos.	EBM Ziffer	Leistung (SLIT)	Leistung (€)	N	Gesamt (€)	N	Gesamt (€)	N	Gesamt (€)
				**Q1**		**Q2-4**		**Q5-12**	
1	09211	Grundpauschale 6.- 59. Lebensjahr	23,10	1	23,10	1	23,10	1	23,10
2	09220	Zuschlag für die HNO Grundversorgung	3,04	0	0,00	1	3,04	1	3,04
3	09222	Zuschlag zu der GOP 09220	0,79	0	0,00	1	0,79	1	0,79
4	30100	Anamnese/Beratung	7,32	4	29,28	0	0,00	1	7,32
5	30111	Diagnostik	24,79	1	24,79	0	0,00	0	0,00
6	40351	Kostenpauschale Sachkosten	5,50	1	5,50	0	0,00	0	0,00
7	30120	Rhinomanometr. Provokationstest	7,44	1	7,44	0	0,00	0	0,00
**EBM-Vergütung RLV (Summe Pos 1,2,4,5,7)**					**84,60**		**26,14**		**33,46**
**Pauschalen und extrabudgetäre Leistungen (Summe Pos. 3,6 und evtl. FL)**					**38,50**		**0,79**		**0,79**
Durchschnittlicher RLV-Fallwert					25,63		25,63		25,63
					**Gesamt (€)**		**Gesamt (€)**		**Gesamt (€)**
AV RLV - Überschreitung zum durchschnittlichen Fallwert (DF) mit 15% berechnet					34,48		25,71		26,80
Pauschalen und extrabudgetäre Leistungen (Summe Pos. 3,6 und evtl. FL)					38,50		0,79		0,79
**Gesamthonorar**					**72,98**		**26,49**		**27,59**
DHPB in Q1, Q2-Q4 bzw. Q1-Q4 2022 (26,28–30)					46,99		46,20		46,40

Tabelle S30: Beispielhafte Berechnung des Honorars eines HNO-Arztes für allergologische Diagnostik und SLIT in 2022 in der KVNo. Der DF wurde zur Berechnung der abgesenkten RLV-Vergütung verwendet. Zum Vergleich der Honorare ist auch das DHPB aufgeführt.

Honorar-Übersicht von Diagnostik und dreijähriger AIT in der KVNo

Anzahl Quartale	Quartale	DHPB (€)	Vergütung in einer budgeteinhaltenden Praxis (€)		Vergütung in einer budgetüberschreitenden Praxis (€)	
			**Diagnostik (nur in Q1) und SCIT**	**Diagnostik (nur in Q1) und SLIT**	**Diagnostik (nur in Q1) und SCIT**	**Diagnostik (nur in Q1) und SLIT**
1	Q1	46,99	183,19	123,10	96,10	72,98
3	Q2-4	46,2	73,85	26,93	44,11	26,49
8	Q5-12	46,4	81,17	34,25	45,21	27,59
**12**	**Q1-12**	**556,79**	**1054,10**	**477,87**	**590,14**	**373,21**

Tabelle S31: Darstellung des maximal erhältlichen und des abgesenkten Honorars (gesamt und pro Quartal) eines HNO-Arztes für allergologische Diagnostik und eine dreijährige AIT in der KVNo bei einem adhärenten Patienten. Zum Vergleich der Honorare ist auch das Quartals- und Gesamt-DHPB dargestellt.

Sachsen

Vergütung der AIT in einer budgetüberschreitenden HNO-Praxis

Pos.	EBM Ziffer	Leistung (SCIT)	Leistung (€)	N	Gesamt (€)	N	Gesamt (€)	N	Gesamt (€)
				Q1		Q2-4		Q5-12	
1	09211	Grundpauschale 6.- 59. Lebensjahr	23,10	1	23,10	1	23,10	1	23,10
2	09220	Zuschlag für die HNO Grundversorgung	3,04	0	0,00	1	3,04	1	3,04
3	09222	Zuschlag zu der GOP 09220	0,79	0	0,00	1	0,79	1	0,79
4	30100	Anamnese/Beratung	7,32	4	29,28	0	0,00	1	7,32
5	30111	Diagnostik	24,79	1	24,79	0	0,00	0	0,00
6	40351	Kostenpauschale Sachkosten	5,50	1	5,50	0	0,00	0	0,00
7	30120	Rhinomanometr. Provokationstest	7,44	1	7,44	0	0,00	0	0,00
8	30130	Injektion Grund- und Fortsetzungs-behandlung	11,49	3	34,47	3	34,47	3	34,47
9	30131	Zuschlag für die 2. Injektion	9,01	1	9,01	0	0,00	0	0,00
**EBM-Vergütung RLV (Summe Pos. 1,2)**					**23,10**		**26,14**		**26,14**
**EBM-Vergütung QZV Diagnostik (Summe Pos. 4,5,7)**					**61,50**		**0,00**		**7,32**
**EBM-Vergütung QZV Hyposensibilisierung (Summe Pos. 8,9)**					**43,49**		**34,47**		**34,47**
**Pauschalen und extrabudgetäre Leistungen (Summe Pos. 3,6 und evtl. FL)**					**5,50**		**0,79**		**0,79**
Durchschnittlicher RLV-Fallwert					25,88		25,88		25,88
Durchschnittlicher QZV Diagnostik-Fallwert					41,06		41,06		41,06
Durchschnittlicher QZV Hyposens.-Fallwert					26,15		26,15		26,15
					**Gesamt (€)**		**Gesamt (€)**		**Gesamt (€)**
AV RLV - Überschreitung zum DF mit 15% berechnet					23,10		25,92		25,92
AV QZV Diagnostik - Überschreitung zum DF mit 15% berechnet					44,13		0,00		7,32
AV QZV Hyposens. - Überschreitung zum DF mit 15% berechnet					28,75		27,40		27,40
Pauschalen und extrabudgetäre Leistungen (Summe Pos. 3,6 und evtl. FL)					5,50		0,79		0,79
**Gesamthonorar**					**101,47**		**54,11**		**61,43**
DHPB in Q1, Q2-Q4 bzw. Q1-Q4 2022 (26,28–30)					53,35		51,93		52,28

Tabelle S32: Beispielhafte Berechnung des Honorars eines HNO-Arztes für allergologische Diagnostik und SCIT in 2022 in der KVS. Der DF wurde zur Berechnung der abgesenkten RLV-Vergütung verwendet. Zum Vergleich der Honorare ist auch das DHPB aufgeführt.

Pos.	EBM Ziffer	Leistung (SLIT)	Leistung (€)	N	Gesamt (€)	N	Gesamt (€)	N	Gesamt (€)
				Q1		Q2-4		Q5-12	
1	09211	Grundpauschale 6.- 59. Lebensjahr	23,10	1	23,10	1	23,10	1	23,10
2	09220	Zuschlag für die HNO Grundversorgung	3,04	0	0,00	1	3,04	1	3,04
3	09222	Zuschlag zu der GOP 09220	0,79	0	0,00	1	0,79	1	0,79
4	30100	Anamnese/Beratung	7,32	4	29,28	0	0,00	1	7,32
5	30111	Diagnostik	24,79	1	24,79	0	0,00	0	0,00
6	40351	Kostenpauschale Sachkosten	5,50	1	5,50	0	0,00	0	0,00
7	30120	Rhinomanometr. Provokationstest	7,44	1	7,44	0	0,00	0	0,00
**EBM-Vergütung RLV (Summe Pos. 1,2)**					**23,10**		**26,14**		**26,14**
**EBM-Vergütung QZV Diagnostik (Summe Pos. 4,5,7)**					**61,50**		**0,00**		**7,32**
**Pauschalen und extrabudgetäre Leistungen (Summe Pos. 3,6 und evtl. FL)**					**5,50**		**0,79**		**0,79**
Durchschnittlicher RLV-Fallwert					25,88		25,88		25,88
Durchschnittlicher QZV Diagnostik-Fallwert					41,06		41,06		41,06
					**Gesamt (€)**		**Gesamt (€)**		**Gesamt (€)**
AV RLV - Überschreitung zum DF mit 15% berechnet					23,10		25,92		25,92
AV QZV Diagnostik - Überschreitung zum DF mit 15% berechnet					44,13		0,00		7,32
Pauschalen und extrabudgetäre Leistungen (Summe Pos. 3,6 und evtl. FL)					5,50		0,79		0,79
**Gesamthonorar**					**72,73**		**26,71**		**34,03**
DHPB in Q1, Q2-Q4 bzw. Q1-Q4 2022 (26,28–30)					53,35		51,93		52,28

Tabelle S33: Beispielhafte Berechnung des Honorars eines HNO-Arztes für allergologische Diagnostik und SLIT in 2022 in der KVS. Der DF wurde zur Berechnung der abgesenkten RLV-Vergütung verwendet. Zum Vergleich der Honorare ist auch das DHPB aufgeführt.

Honorar-Übersicht von Diagnostik und dreijähriger AIT in der KVS

Anzahl Quartale	Quartale	DHPB (€)	Vergütung in einer budgeteinhaltenden Praxis (€)		Vergütung in einer budgetüberschreitenden Praxis (€)	
			Diagnostik (nur in Q1) und SCIT	Diagnostik (nur in Q1) und SLIT	Diagnostik (nur in Q1) und SCIT	Diagnostik (nur in Q1) und SLIT
1	Q1	53,35	133,58	90,10	101,47	72,73
3	Q2-4	51,93	61,40	26,93	54,11	26,71
8	Q5-12	52,28	68,72	34,25	61,43	34,03
GOP 99675A und 99675B			60,00		60,00	
**12**	**Q1-12**	**627,38**	**927,58**	**444,87**	**815,22**	**425,09**

Tabelle S34: Darstellung des maximal erhältlichen und des abgesenkten Honorars (gesamt und pro Quartal) eines HNO-Arztes für allergologische Diagnostik und eine dreijährige AIT in der KVS bei einem adhärenten Patienten. Zum Vergleich der Honorare ist auch das Quartals- und Gesamt-DHPB dargestellt.

Sachsen-Anhalt

Vergütung der AIT in einer budgetüberschreitenden HNO-Praxis

Pos.	EBM Ziffer	Leistung (SCIT)	Leistung (€)	N	Gesamt (€)	N	Gesamt (€)	N	Gesamt (€)
				Q1		Q2-4		Q5-12	
1	09211	Grundpauschale 6.- 59. Lebensjahr	23,10	1	23,10	1	23,10	1	23,10
2	09220	Zuschlag für die HNO Grundversorgung	3,04	0	0,00	1	3,04	1	3,04
3	09222	Zuschlag zu der GOP 09220	0,79	0	0,00	1	0,79	1	0,79
4	30100	Anamnese/Beratung	7,32	4	29,28	0	0,00	1	7,32
5	30111	Diagnostik	24,79	1	24,79	0	0,00	0	0,00
6	40351	Kostenpauschale Sachkosten	5,50	1	5,50	0	0,00	0	0,00
7	30120	Rhinomanometr. Provokationstest	7,44	1	7,44	0	0,00	0	0,00
8	30130	Injektion Grund- und Fortsetzungs-behandlung	11,49	3	34,47	3	34,47	3	34,47
9	30131	Zuschlag für die 2. Injektion	9,01	1	9,01	0	0,00	0	0,00
**EBM-Vergütung RLV (Summe Pos. 1,2)**					**23,10**		**26,14**		**26,14**
**EBM-Vergütung QZV Diagnostik (Summe Pos. 4,5,7)**					**61,50**		**0,00**		**7,32**
**EBM-Vergütung QZV Hyposensibilisierung (Summe Pos. 8,9)**					**43,49**		**34,47**		**34,47**
**Pauschalen und extrabudgetäre Leistungen (Summe Pos. 3,6 und evtl. FL)**					**5,50**		**0,79**		**0,79**
Durchschnittlicher RLV-Fallwert					27,14		27,14		27,14
Durchschnittlicher QZV Diagnostik-Fallwert					27,45		27,45		27,45
Durchschnittlicher QZV Hyposens.-Fallwert					22,05		22,05		22,05
					**Gesamt (€)**		**Gesamt (€)**		**Gesamt (€)**
AV RLV - Überschreitung zum DF mit 15% berechnet					23,10		26,14		26,14
AV QZV Diagnostik - Überschreitung zum DF mit 15% berechnet					32,56		0,00		7,32
AV QZV Hyposens. - Überschreitung zum DF mit 15% berechnet					25,27		23,91		23,91
Pauschalen und extrabudgetäre Leistungen (Summe Pos. 3,6 und evtl. FL)					5,50		0,79		0,79
**Gesamthonorar**					**86,42**		**50,84**		**58,16**
DHPB in Q1, Q2-Q4 bzw. Q1-Q4 2022 (26,28–30)					49,15		49,41		49,35

Tabelle S35: Beispielhafte Berechnung des Honorars eines HNO-Arztes für allergologische Diagnostik und SCIT in 2022 in der KVSA. Der DF wurde zur Berechnung der abgesenkten RLV-Vergütung verwendet. Zum Vergleich der Honorare ist auch das DHPB aufgeführt.

Pos.	EBM Ziffer	Leistung (SLIT)	Leistung (€)	N	Gesamt (€)	N	Gesamt (€)	N	Gesamt (€)
				Q1		Q2-4		Q5-12	
1	09211	Grundpauschale 6.- 59. Lebensjahr	23,10	1	23,10	1	23,10	1	23,10
2	09220	Zuschlag für die HNO Grundversorgung	3,04	0	0,00	1	3,04	1	3,04
3	09222	Zuschlag zu der GOP 09220	0,79	0	0,00	1	0,79	1	0,79
4	30100	Anamnese/Beratung	7,32	4	29,28	0	0,00	1	7,32
5	30111	Diagnostik	24,79	1	24,79	0	0,00	0	0,00
6	40351	Kostenpauschale Sachkosten	5,50	1	5,50	0	0,00	0	0,00
7	30120	Rhinomanometr. Provokationstest	7,44	1	7,44	0	0,00	0	0,00
**EBM-Vergütung RLV (Summe Pos. 1,2)**					**23,10**		**26,14**		**26,14**
**EBM-Vergütung QZV Diagnostik (Summe Pos. 4,5,7)**					**61,50**		**0,00**		**7,32**
**Pauschalen und extrabudgetäre Leistungen (Summe Pos. 3,6 und evtl. FL)**					**5,50**		**0,79**		**0,79**
Durchschnittlicher RLV-Fallwert					27,14		27,14		27,14
Durchschnittlicher QZV Diagnostik-Fallwert					27,45		27,45		27,45
					**Gesamt (€)**		**Gesamt (€)**		**Gesamt (€)**
AV RLV - Überschreitung zum DF mit 15% berechnet					23,10		26,14		26,14
AV QZV Diagnostik - Überschreitung zum DF mit 15% berechnet					32,56		0,00		7,32
Pauschalen und extrabudgetäre Leistungen (Summe Pos. 3,6 und evtl. FL)					5,50		0,79		0,79
**Gesamthonorar**					**61,15**		**26,93**		**34,25**
DHPB in Q1, Q2-Q4 bzw. Q1-Q4 2022 (26,28–30)					49,15		49,41		49,35

Tabelle S36: Beispielhafte Berechnung des Honorars eines HNO-Arztes für allergologische Diagnostik und SLIT in 2022 in der KVSA. Der DF wurde zur Berechnung der abgesenkten RLV-Vergütung verwendet. Zum Vergleich der Honorare ist auch das DHPB aufgeführt.

Honorar-Übersicht von Diagnostik und dreijähriger AIT in der KVSA

Anzahl Quartale	Quartale	DHPB (€)	Vergütung in einer budgeteinhaltenden Praxis (€)		Vergütung in einer budgetüberschreitenden Praxis (€)	
			Diagnostik (nur in Q1) und SCIT	Diagnostik (nur in Q1) und SLIT	Diagnostik (nur in Q1) und SCIT	Diagnostik (nur in Q1) und SLIT
1	Q1	49,15	133,58	90,10	86,42	61,15
3	Q2-4	49,41	61,40	26,93	50,84	26,93
8	Q5-12	49,35	68,72	34,25	58,16	34,25
**12**	**Q1-12**	**592,18**	**867,58**	**444,87**	**704,24**	**415,93**

Tabelle S37: Darstellung des maximal erhältlichen und des abgesenkten Honorars (gesamt und pro Quartal) eines HNO-Arztes für allergologische Diagnostik und eine dreijährige AIT in der KVSA bei einem adhärenten Patienten. Zum Vergleich der Honorare ist auch das Quartals- und Gesamt-DHPB dargestellt.

Westfalen-Lippe

Vergütung der AIT in einer budgetüberschreitenden HNO-Praxis

Pos.	EBM Ziffer	Leistung (SCIT)	Leistung (€)	N	Gesamt (€)	N	Gesamt (€)	N	Gesamt (€)
				Q1		Q2-4		Q5-12	
1	09211	Grundpauschale 6.- 59. Lebensjahr	23,10	1	23,10	1	23,10	1	23,10
2	09220	Zuschlag für die HNO Grundversorgung	3,04	0	0,00	1	3,04	1	3,04
3	09222	Zuschlag zu der GOP 09220	0,79	0	0,00	1	0,79	1	0,79
4	30100	Anamnese/Beratung	7,32	4	29,28	0	0,00	1	7,32
5	30111	Diagnostik	24,79	1	24,79	0	0,00	0	0,00
6	40351	Kostenpauschale Sachkosten	5,50	1	5,50	0	0,00	0	0,00
7	30120	Rhinomanometr. Provokationstest	7,44	1	7,44	0	0,00	0	0,00
8	30130	Injektion Grund- und Fortsetzungs-behandlung	11,49	3	34,47	3	34,47	3	34,47
9	30131	Zuschlag für die 2. Injektion	9,01	1	9,01	0	0,00	0	0,00
**EBM-Vergütung RLV (Summe Pos 1,2,4,5,7,8,9)**					**128,08**		**60,61**		**67,93**
**Pauschalen und extrabudgetäre Leistungen (Summe Pos. 3,6 und evtl. FL)**					**5,50**		**0,79**		**0,79**
Durchschnittlicher RLV-Fallwert					31,34		31,34		31,34
					**Gesamt (€)**		**Gesamt (€)**		**Gesamt (€)**
AV RLV - Überschreitung zum durchschnittlichen Fallwert (DF) mit 15% berechnet					45,85		35,73		36,83
Pauschalen und extrabudgetäre Leistungen (Summe Pos. 3,6 und evtl. FL)					5,50		0,79		0,79
**Gesamthonorar**					**51,35**		**36,52**		**37,62**
DHPB in Q1, Q2-Q4 bzw. Q1-Q4 2022 (26,28–30)					48,19		46,79		47,14

Tabelle S38: Beispielhafte Berechnung des Honorars eines HNO-Arztes für allergologische Diagnostik und SCIT in 2022 in der KVWL. Der DF wurde zur Berechnung der abgesenkten RLV-Vergütung verwendet. Zum Vergleich der Honorare ist auch das DHPB aufgeführt.

Pos.	EBM Ziffer	Leistung (SLIT)	Leistung (€)	N	Gesamt (€)	N	Gesamt (€)	N	Gesamt (€)
				Q1		Q2-4		Q5-12	
1	09211	Grundpauschale 6.- 59. Lebensjahr	23,10	1	23,10	1	23,10	1	23,10
2	09220	Zuschlag für die HNO Grundversorgung	3,04	0	0,00	1	3,04	1	3,04
3	09222	Zuschlag zu der GOP 09220	0,79	0	0,00	1	0,79	1	0,79
4	30100	Anamnese/Beratung	7,32	4	29,28	0	0,00	1	7,32
5	30111	Diagnostik	24,79	1	24,79	0	0,00	0	0,00
6	40351	Kostenpauschale Sachkosten	5,50	1	5,50	0	0,00	0	0,00
7	30120	Rhinomanometr. Provokationstest	7,44	1	7,44	0	0,00	0	0,00
**EBM-Vergütung RLV (Summe Pos 1,2,4,5,7)**					**84,60**		**26,14**		**33,46**
**Pauschalen und extrabudgetäre Leistungen (Summe Pos. 3,6 und evtl. FL)**					**5,50**		**0,79**		**0,79**
Durchschnittlicher RLV-Fallwert					31,34		31,34		31,34
					**Gesamt (€)**		**Gesamt (€)**		**Gesamt (€)**
AV RLV - Überschreitung zum durchschnittlichen Fallwert (DF) mit 15% berechnet					39,33		26,14		31,66
Pauschalen und extrabudgetäre Leistungen (Summe Pos. 3,6 und evtl. FL)					5,50		0,79		0,79
**Gesamthonorar**					**44,83**		**26,93**		**32,45**
DHPB in Q1, Q2-Q4 bzw. Q1-Q4 2022 (26,28–30)					48,19		46,79		47,14

Tabelle S39: Beispielhafte Berechnung des Honorars eines HNO-Arztes für allergologische Diagnostik und SLIT in 2022 in der KVWL. Der DF wurde zur Berechnung der abgesenkten RLV-Vergütung verwendet. Zum Vergleich der Honorare ist auch das DHPB aufgeführt.

Honorar-Übersicht von Diagnostik und dreijähriger AIT in der KVWL

Anzahl Quartale	Quartale	DHPB (€)	Vergütung in einer budgeteinhaltenden Praxis (€)		Vergütung in einer budgetüberschreitenden Praxis (€)	
			Diagnostik (nur in Q1) und SCIT	Diagnostik (nur in Q1) und SLIT	Diagnostik (nur in Q1) und SCIT	Diagnostik (nur in Q1) und SLIT
1	Q1	48,19	133,58	90,10	51,35	44,83
3	Q2-4	46,79	61,40	26,93	36,52	26,93
8	Q5-12	47,14	68,72	34,25	37,62	32,45
**12**	**Q1-12**	**565,68**	**867,58**	**444,87**	**461,85**	**385,18**

Tabelle S40: Darstellung des maximal erhältlichen und des abgesenkten Honorars (gesamt und pro Quartal) eines HNO-Arztes für allergologische Diagnostik und eine dreijährige AIT in der KVWL bei einem adhärenten Patienten. Zum Vergleich der Honorare ist auch das Quartals- und Gesamt-DHPB dargestellt.

Deutschlandweite Honorierung von Diagnostik und dreijähriger AIT in budgetüberschreitenden HNO-Praxen

	Gesamthonorar 3 Jahre (€)				
**KV**	**DHPB**	**Diagnostik (nur in Q1) und SCIT**	**Differenz zum DHPB**	**Diagnostik (nur in Q1) und SLIT**	**Differenz zum DHPB**
Baden-Württemberg	598,41	592,79	–5,62	402,51	–195,90
Bayern	683,75	716,67	32,92	419,62	–264,13
Berlin	622,67	612,98	–9,69	545,57	–77,10
Brandenburg	581,66	610,64	28,98	358,02	–223,64
Bremen	584,85	622,21	37,36	362,77	–222,08
Hamburg	696,72	825,35	128,63	373,09	–323,63
Hessen	628,08	707,04	78,96	427,37	–200,71
Niedersachsen	604,21	562,69	–41,52	392,28	–211,93
Nordrhein	556,79	590,14	33,35	373,21	–183,58
Sachsen	627,38	815,22	187,84	425,09	–202,29
Sachsen-Anhalt	592,18	704,24	112,06	415,93	–176,25
Westfalen-Lippe	565,68	461,85	–103,83	385,18	–180,50

Tabelle S41: Beispielhafte Berechnung des abgesenkten Gesamthonorars eines HNO-Arztes für allergologische Diagnostik und eine dreijährige AIT in zahlreichen KV bei einem adhärenten Patienten auf Basis von Zahlen von 2022.

Vergleich der Stunden-Honorierung bei SCIT und SLIT in einer budgeteinhaltenden Praxis

Das Stundenhonorar ergibt sich aus dem Verhältnis der Honorierung zum zeitlichen Aufwand. Legt man die Prüfzeiten aus dem EBM (2) der Zeitrechnung zugrunde, ergeben sich für alle Leistungen im Q1 50 Minuten für die SCIT. In der Fortsetzungsbehandlung stehen jeweils 22 bzw. 27 Minuten in Q2-4 bzw. in Q5-12 zur Verfügung. Die Prüfzeiten bei SLIT betragen je nach Quartal jeweils 39, 13 bzw. 18 Minuten (siehe Tabelle S4). Im Folgenden wird auf die Q5-12 fokussiert, als diejenigen mit dem niedrigsten Stundenhonorar, um das Mindeststundenhonorar zu ermitteln.

Die Honorare pro Fall (H_F) mit einer SCIT in Q5-12 für die zwölf untersuchten KV entnimmt man der Tabelle „Honorar-Übersicht von Diagnostik und dreijähriger AIT“ der jeweiligen KV und sind in Tabelle S42 dargestellt. Das Stundenhonorar (H_h) ergibt sich aus dem Verhältnis des H_F zur investierten Prüfzeit (t_P) und anschließender Multiplikation mit dem Umrechnungsfaktor 60, da eine Stunde 60 Minuten umfasst, entsprechend folgender Formel: $H_h =. \frac{H_F}{t_P} \text{ x } 60$.

Um die Wirtschaftlichkeit zu beurteilen, werden dem Stundenhonorar die Aufwendungen von 80,47 €/h gegenübergestellt (siehe Methoden). Das ergibt bei SCIT in einer budgeteinhaltenden HNO-Praxis in der KVB den Netto-Ertrag pro Stunde (siehe Tabelle S42)

Zu beachten ist, dass die hier dargestellten Netto-Erträge pro Stunde nicht mit dem Einkommen der Ärztinnen bzw. Ärzte zu verwechseln sind. Das „Nettoeinkommen“ ergibt sich erst nach Abzug von Steuern, Beiträgen zur Kranken- und Pflegeversicherung sowie zu Versorgungswerken und ggf. weiteren Vorsorgeaufwendungen (27).

	**SCIT in Q5-12**			
**KV**	**Honorar pro Fall (H_F) (€)**	**Honorar pro Stunde (H_h) (€)**	**Aufwendungen pro Stunde (€)**	**Netto-Ertrag pro Stunde (€)**
KVBaWü	77,72	172,71	80,47	92,24
KVB	72,77	161,71	80,47	81,24
KVBerlin	77,90	173,11	80,47	92,64
KVBB	83,72	186,04	80,47	105,57
KVHB	78,74	174,98	80,47	94,51
KVHH	69,83	155,18	80,47	74,71
KVH	72,08	160,19	80,47	79,72
KVNS	68,72	152,71	80,47	72,24
KVNo	81,17	180,38	80,47	99,91
KVS	68,72	152,71	80,47	72,24
KVSA	68,72	152,71	80,47	72,24
KVWL	68,72	152,71	80,47	72,24

Tabelle S42: Beispielhafte Berechnung der Netto-Stundenerträge für SCIT in Q5-12 in zahlreichen KV nach Abzug von Aufwendungen. Das Honorar/Fall (H_F) entnimmt man der Tabelle „Honorar-Übersicht von Diagnostik und dreijähriger AIT“ der jeweiligen KV. Bei einer Prüfzeit (t_P) von 27 Min. in Q5-12 errechnet sich das -Stundenhonorar (H_h) folgendermaßen: : $H_h =. \frac{H_F}{27} \text{ x } 60$. Der Netto-Ertrag pro Stunde ergibt sich aus der Subtraktion der Aufwendungen pro Stunde vom H_h.

Die Berechnung des Netto-Ertrags pro Stunde für die SLIT in Q5-12 wird entsprechend der obigen Rechnung durchgeführt (Verwendung einer Prüfzeit von 18 Minuten). Werden bei den Aufwendungen

die Personalkosten (PK) berücksichtigt, ergibt sich ein Netto-Ertrag pro Stunde von 33,70 € für alle KV mit Ausnahme der KVBerlin bzw. der KVHH (siehe Tabelle S43), in der die SLIT mit der SNR 30130T zusätzlich gefördert wird bzw. Garantiequoten als Instrument für die Honorierung verwendet werden (siehe Tabelle S2).

Der personelle Aufwand in einer Praxis bei SLIT ist generell überschaubar, so wurde der Einfachheit halber zusätzlich eine Modellberechnung für das Stundenhonorar bei SLIT ohne Berücksichtigung jeglicher Personalkosten durchgeführt. Dem Stundenhonorar wurden nur die Aufwendungen für z.B. Material, Labor, Miete und die Nebenkosten für Praxisräume in Höhe von insgesamt 34,60 € vom abgezogen (43% der Gesamtaufwendungen; siehe Methoden). Das ergibt bei einer SLIT in einer budgeteinhaltenden HNO-Praxis einen Netto-Ertrag vor Abzügen von 79,57 €/h in den meisten KV (siehe Tabelle S43).

Der höheren Honorierung bei SCIT steht im Vergleich zur SLIT ein höherer personeller Mehraufwand gegenüber. Werden daher bei SLIT die Personalkosten nicht berücksichtigt, ergeben sich vergleichbare Netto-Erträge pro Stunde für beide Applikationsformen (siehe Tabellen S42 und S43). Die Höhe der Honorierung für das Personal bei SCIT in den jeweiligen KV variiert in dieser Modellberechnung zwischen 24,51 €/h (KVBerlin) und 71,87 €/h (KV Brandenburg) (siehe Tabelle S44).

	**SLIT in Q5-12**					
**KV**	**Honorar pro Fall (H_F) (€)**	**Honorar pro Stunde (H_h) (€)**	**Aufwendungen pro Stunde (€)**		**Netto-Ertrag pro Stunde (€)**	
			**Inkl. PK**	**Exkl. PK**	**Inkl. PK**	**Exkl. PK**
KVBaWü	34,25	114,17	80,47	34,60	33,70	79,57
KVB	34,25	114,17	80,47	34,60	33,70	79,57
KVBerlin	44,58	148,60	80,47	34,60	68,13	114,00
KVBB	34,25	114,17	80,47	34,60	33,70	79,57
KVHB	34,25	114,17	80,47	34,60	33,70	79,57
KVHH	34,79	115,97	80,47	34,60	35,50	81,37
KVH	34,25	114,17	80,47	34,60	33,70	79,57
KVNS	34,25	114,17	80,47	34,60	33,70	79,57
KVNo	34,25	114,17	80,47	34,60	33,70	79,57
KVS	34,25	114,17	80,47	34,60	33,70	79,57
KVSA	34,25	114,17	80,47	34,60	33,70	79,57
KVWL	34,25	114,17	80,47	34,60	33,70	79,57

Tabelle S43: Beispielhafte Berechnung der Netto-Stundenerträge für SLIT in Q5-12 in zahlreichen KV nach Abzug von Aufwendungen. Das Honorar/Fall (H_F) entnimmt man der Tabelle „Honorar-Übersicht von Diagnostik und dreijähriger AIT“ der jeweiligen KV. Bei einer Prüfzeit (t_P) von 18 Min. in Q5-12 errechnet sich das -Stundenhonorar (H_h) folgendermaßen: : $H_h = . \frac{H_F}{18} \times 60$. Der Netto-Ertrag pro Stunde ergibt sich aus der Subtraktion der Aufwendungen pro Stunde vom H_h. Die Berechnungen wurden unter Berücksichtigung der Aufwendungen pro Stunde mit oder ohne Personalkosten (PK) durchgeführt.

	**Netto-Ertrag pro Stunde in Q5-12 (€)**		
**KV**	**SCIT**	**SLIT**	**Differenz SCIT-SLIT**
KVBaWü	92,24	33,70	58,54
KVB	81,24	33,70	47,54
KVBerlin	92,64	68,13	24,51
KVBB	105,57	33,70	71,87
KVHB	94,51	33,70	60,81
KVHH	74,71	35,50	39,21
KVH	79,72	33,70	46,02
KVNS	72,24	33,70	38,54
KVNo	99,91	33,70	66,21
KVS	72,24	33,70	38,54
KVSA	72,24	33,70	38,54
KVWL	72,24	33,70	38,54

Tabelle S44: Beispielhafte Berechnung der Netto-Stundenerträge eines HNO-Arztes für SCIT und SLIT in Q5-12 in zahlreichen KV nach Abzug von Aufwendungen. Die Differenz kann als die Honorierung für den personellen Mehraufwand der SCIT im Vergleich zur SLIT betrachtet werden.

ABKÜRZUNGSVERZEICHNIS

AA: Allergisches Asthma

AIT: Allergenspezifische Immuntherapie; allergen-specific immunotherapy

ALK: Altersklasse

AR: Allergische Rhinitis

AV: Abgesenkte Vergütung

DF: Durchschnittlicher Fallwert

DHPB: durchschnittliches Honorar pro Behandlungsfall

EBM: Einheitlicher Bewertungsmaßstab

ENT: Ear, nose, and throat physician

FL: Förderungswürdige Leistungen

GOP: Gebührenordnungsposition

h: Stunde

HNO: Hals-Nasen-Ohren-Arzt

H_F: Honorar pro Fall

H_h: Stundenhonorar

KV: Kassenärztliche Vereinigung

KVB: Kassenärztliche Vereinigung Bayern

KVBerlin: Kassenärztliche Vereinigung Berlin

KVBaWü: Kassenärztliche Vereinigung Baden-Württemberg

KVH: Kassenärztliche Vereinigung Hessen

KVMV: Kassenärztliche Vereinigung Mecklenburg-Vorpommern

KVN: Kassenärztliche Vereinigung Niedersachsen

KVNo: Kassenärztliche Vereinigung Nordrhein,

KVRP: Kassenärztliche Vereinigung Rheinland-Pfalz

KVS: Kassenärztliche Vereinigung Sachsen

KVSA: Kassenärztliche Vereinigung Sachsen-Anhalt

KVSaarland: Kassenärztliche Vereinigung Saarland

KVSH: Kassenärztliche Vereinigung Schleswig-Holstein

KVT: Kassenärztliche Vereinigung Thüringen

KVWL: Kassenärztliche Vereinigung Westfalen-Lippe

Min: Minuten

MGV: morbiditätsbedingten Gesamtvergütung

N: Anzahl

OPW: Orientierungspunktwert

PK: Personalkosten

Pos.: Position

Q1: Quartal 1; erstes Quartal einer AIT

Q2-4: Quartale 2 bis 4; zweites bis viertes Quartal einer AIT

Q5-12: Quartale 5 bis 12; fünftes bis zwölftes Quartal einer AIT

QZV: Qualitätsgebundenes Zusatzvolumen

RLV: Regelleistungsvolumen

RWE: Real-World Evidence

SCIT: Subkutane Immuntherapie

SNR: Symbolnummer.

SLIT: sublinguale Immuntherapie

t_P: Prüfzeit

ZiPP: Zi-Praxis-Panels (Praxis-Panel des Zentralinstituts für die kassenärztliche Versorgung in der Bundesrepublik Deutschland)

QUELLEN:

1. Klimek L. Honorierung allergologischer Leistungen weiterhin uneinheitlich. Allergo J. 2022;31(8):53--55.

2. Kassenärztliche Bundesvereinigung KdöR. Online-Version des EBM [Internet]. Kassenärztliche Bundesvereinigung (KBV); 2023 [zitiert 21. Dezember 2023]. Verfügbar unter: https://www.kbv.de/html/online-ebm.php

3. Kassenärztliche Vereinigung Rheinland-Pfalz. Honorarverteilungsmaßstab (HVM) zum 1. Januar 2022 [Internet]. 2021 [zitiert 12. Februar 2024]. Verfügbar unter: https://www.kv-rlp.de/fileadmin/user_upload/Downloads/Mitglieder/Verguetung/Honorar/Honorarverteilung/2022_Q1/HVM_ab_01-01-22.pdf

4. Kassenärztliche Vereinigung Schleswig-Holstein. Honorarverteilungsmaßstab der KVSH [Internet]. 2021 [zitiert 12. Februar 2024]. Verfügbar unter: https://www.kvsh.de/fileadmin/user_upload/dokumente/Praxis/Rechtsvorschriften/HVM/HVM_ab_01012022_Beschluss_AV_17112021.pdf

5. Kassenärztlichen Vereinigung Saarland. Honorarverteilungsmaßstab der Kassenärztlichen Vereinigung Saarland [Internet]. 2021 [zitiert 12. Februar 2024]. Verfügbar unter: https://www.kvsaarland.de/wp-content/uploads/2023/01/1b_HVM-gueltig-ab-01.01.2022.pdf

6. Kassenärztlichen Vereinigung Thüringen (KVT). Honorarverteilungsmaßstab (HVM) der Kassenärztlichen Vereinigung Thüringen (KVT) [Internet]. 2022 [zitiert 12. Februar 2024]. Verfügbar unter: https://www.kv-thueringen.de/fileadmin/media2/Abrechnung/4300/4300_HVM/4300_HVM_2022/LF_nach_VV_10092022_I_2022.pdf

7. Kassenärztliche Vereinigung Baden-Württemberg. Archiv Arzthonorar [Internet]. Verfügbar unter: https://www.kvbawue.de/praxis/abrechnung-honorar/arzthonorare/archiv-arzthonorare

8. Kassenärztliche Vereinigung Bayern. Fallwerte und Quoten [Internet]. Verfügbar unter: https://www.kvb.de/mitglieder/abrechnung/honorar/fallwerte-und-quoten

9. Kassenärztliche Vereinigung Bayern. Honorarverträge [Internet]. Verfügbar unter: https://www.kvb.de/mitglieder/abrechnung/verguetungsvertraege/honorarvertraege

10. Kassenärztliche Vereinigung Berlin. RLV, QZV, Quartalspunktwerte (Archiv) [Internet]. Verfügbar unter: https://www.kvberlin.de/fuer-praxen/alles-fuer-den-praxisalltag/abrechnung-/-honorar/honorarverteilung/rlv-qzv-punktwerte-archiv

11. Kassenärztliche Vereinigung Berlin. Honorarvertrag [Internet]. Verfügbar unter: https://www.kvberlin.de/fuer-praxen/alles-fuer-den-praxisalltag/vertrage-und-recht/vertraege/honorarvertraege

12. Kassenärztliche Vereinigung Brandenburg. Honorar - Honorarverteilungsmaßstab / Fallwerte und Durchschnittsfallzahlen [Internet]. Verfügbar unter: https://www.kvbb.de/praxis/praxiswissen/abrechnung-honorar/honorar

13. Kassenärztliche Vereinigung Bremen. RLV: Durchschnittliche Fallwerte und Fallzahlen [Internet]. Verfügbar unter: https://www.kvhb.de/download-item-detail//rlv-durchschnittliche-fallwerte-und-fallzahlen

14. Kassenärztliche Vereinigung Bremen. EBM: Regionale Pseudonummern-Gebührenordnung [Internet]. Verfügbar unter: https://www.kvhb.de/download-item-detail//ebm-regionale-pseudonummern-gebuehrenordnung

15. Kassenärztliche Vereinigung Hamburg. Honorarvereinbarung 2022 [Internet]. Verfügbar unter: https://www.kvhh.net/_Resources/Persistent/6/7/4/1/67411b540d7d9321f1d8775ab4b6de3c0c447e1f/hon2022%205.nt_lesefassung_2023-03-22.pdf

16. Kassenärztliche Vereinigung Hamburg. Garantiequoten [Internet]. Verfügbar unter: https://www.kvhh.net/de/praxis/abrechnung-and-honorar/garantiequoten.html

17. Kassenärztliche Vereinigung Hessen. Honoraranalysen [Internet]. Verfügbar unter: https://www.kvhessen.de/honorar

18. Kassenärztliche Vereinigung Hessen. Hessenspezifische Gebührenordnungspositionen - Stand: 1. Quartal 2022 [Internet]. Verfügbar unter: https://www.kvhessen.de/fileadmin/user_upload/kvhessen/Mitglieder/Abrechnung_Honorar/Hessen-GOP_2022_Q1.pdf

19. Kassenärztliche Vereinigung Hessen. Honorarverteilungsmaßstab [Internet]. Verfügbar unter: https://www.kvhessen.de/honorar/hvm-archiv

20. Kassenärztliche Vereinigung Niedersachsen. Amtliche Mitteilung der KVN zum HVM: Mindestfallwerte und Fallzahlgrenzen [Internet]. Verfügbar unter: https://www.kvn.de/Mitglieder/Abrechnung_+Honorar+und+Vertrag/Honorarverteilung.html

21. Kassenärztliche Vereinigung Nordrhein. RLV/QZV [Internet]. Verfügbar unter: https://www.kvno.de/praxis/abrechnung-honorar/rlv-qzv

22. AOK PLUS - Die Gesundheitskasse für Sachsen und Thüringen., BKK Landesverband Mitte, IKK classic, KNAPPSCHAFT, Regionaldirektion Chemnitz, Ersatzkassen, Kassenärztlichen Vereinigung Sachsen. Vereinbarung zur Festlegung der regionalen Punktwerte in Sachsen und der sächsischen Gebührenordnung (SGO) zur Festlegung der Gesamtvergütung in Sachsen zur Festlegung des kassenspezifischen Behandlungsbedarfs [Internet]. Verfügbar unter: https://www.kvsachsen.de/fileadmin/api/contracts/MGV_2022_unterzeichnet_scan_compressed.pdf

23. Kassenärztliche Vereinigung Sachsen. Abrechnungsergebnisse [Internet]. Verfügbar unter: https://www.kvsachsen.de/fuer-praxen/honorar-und-abrechnung/honorar/honorardaten/archiv-der-verguetungsrelevanten-daten-und-abrechnungsergebnisse

24. Kassenärztliche Vereinigung Sachsen-Anhalt. Honorarverteilung [Internet]. Verfügbar unter: https://www.kvsa.de/praxis/abrechnung-honorar/honorarverteilung/2022.html

25. Kassenärztliche Vereinigung Westfalen-Lippe. Fallwerte für RLV und QZV [Internet]. Verfügbar unter: https://www.kvwl.de/mitglieder/abrechnung-honorar/honorarverteilung-und-berechnung/fallwerte-fuer-rlv-und-qzv

26. Kassenärztliche Bundesvereinigung KdöR. Honorarbericht - Quartal 1/2022 [Internet]. 2023 [zitiert 18. Dezember 2023]. Verfügbar unter: https://www.kbv.de/media/sp/KBV_Honorarbericht_Q1-2022_web.pdf.

27. Zentralinstitut für die kassenärztliche Versorgung in Deutschland. Zi-Praxis-Panel. Jahresbericht 2022. Wirtschaftliche Situation und Rahmenbedingungen in der vertragsärztlichen Versorgung

der Jahre 2018 bis 2021. [Internet]. 2024 [zitiert 29. April 2024]. Verfügbar unter: https://www.zi-pp.de/pdf/ZiPP_Jahresbericht_2022.pdf

28. Kassenärztliche Bundesvereinigung KdöR. Honorarbericht - Quartal 2/2022 [Internet]. 2023 [zitiert 18. Dezember 2023]. Verfügbar unter: https://www.kbv.de/media/sp/KBV_Honorarbericht_Q2-2022_web.pdf.

29. Kassenärztliche Bundesvereinigung KdöR. Honorarbericht - Quartal 3/2022 [Internet]. 2023 [zitiert 18. Dezember 2023]. Verfügbar unter: https://www.kbv.de/media/sp/KBV_Honorarbericht_Q3-2022_web.pdf.

30. Kassenärztliche Bundesvereinigung KdöR. Honorarbericht - Quartal 4/2022 [Internet]. 2023 [zitiert 20. März 2024]. Verfügbar unter: https://www.kbv.de/media/sp/KBV_Honorarbericht_Q4-2022.pdf.
